# Development of Biginelli-based ZnO-coupled carbomer-gel-coated wound dressing gauze with enhanced antibacterial activity†

**DOI:** 10.1039/d5ra00236b

**Published:** 2025-04-09

**Authors:** Bulle Shah, Narinder Singh, Doo Ok Jang

**Affiliations:** a Department of Chemistry, Indian Institute of Technology Ropar Rupnagar Punjab 140001 India nsingh@iitrpr.ac.in; b Department of Chemistry, Yonsei University Wonju 26493 Republic of Korea dojang@yonsei.ac.kr

## Abstract

A multicomponent Biginelli reaction was used to produce biologically active dihydropyrimidones that were then combined with ZnO nanoparticles. Biginelli compounds synthesized with various alkyl chains were characterized using high-resolution mass spectrometry as well as ^1^H- and ^13^C-NMR spectroscopy. Efficient antibacterial gels were developed by introducing the prepared Biginelli compounds and ZnO nanoparticles into a carbomer polymer matrix. Antibacterial screening revealed that the ABS-G4 gel exhibited the highest antibacterial potential, with minimum inhibitory concentrations of 16 ± 2 and 12 ± 2 μg mL^−1^ against *Escherichia coli* and *Staphylococcus aureus*, respectively. The ABS-G4 gel was characterized using rheological studies, field-emission scanning electron microscopy, energy-dispersive X-ray spectroscopy, powder X-ray diffraction, and atomic force microscopy. The ABS-G4 gel was showing more antibacterial efficacy toward Gram-positive strains of bacteria than Gram-positive ones. An antibacterial dressing was formed by coating the developed gel onto a gauze dressing.

## Abstract 2

A multicomponent Biginelli reaction was used to produce biologically active dihydropyrimidones that were then combined with ZnO nanoparticles.

## Graphical abstract

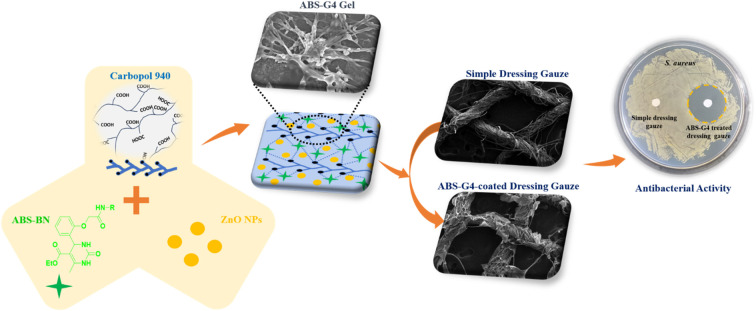

## 1. Introduction

Infections caused by pathogenic bacteria pose significant problems for human life.^1–5^ Cotton fabric is extensively used in a number of applications, including as gauze dressings and sutures in healthcare applications, owing to its softness, biodegradability, and regenerative capability. However, these fabrics are susceptible to microbial contamination, which presents challenges in healthcare applications and highlights the importance of maintaining hygiene and health standards. Several studies have reported the use of various metal coatings, including Ag, Au, and Pt nanoparticles (NPs), for introducing antimicrobial properties into textiles.^6–8^ Nevertheless, these approaches may encounter limitations, such as complexities associated with the coating technique and high coating-material costs.^9^ Hence, textiles with inherent antimicrobial and/or self-cleaning properties are essential for daily use.

Millions of individuals succumb to microbial diseases each year, leading to substantial economic repercussions.^10–13^ The use of antimicrobial coatings is anticipated to diminish transmission, thereby lowering both morbidity and mortality rates and decreasing reliance on antibiotic treatments, among which antibiotic-resistant pathogens present the most challenges, with limited treatment options available.^14–16^ A recent report highlighted the fact that antibiotic-resistant bacteria caused 1.2 million deaths annually.^16^ This antibiotic-resistance phenomenon is often referred to as the “silent pandemic,” which highlights its significant impact on mortality despite receiving minimal attention from the media. The US Centers for Disease Control and Prevention (CDC) released a special report during the COVID-19 pandemic that highlighted the rise in antibiotic-resistant infections and fatalities;^17^ such infections were observed in both hospital settings^18^ and the broader community. Recent research has revealed that healthcare facilities that adopt antimicrobial coatings show significantly improved patient outcomes and notably less environmental contamination.^19^

In a world increasingly challenged by antibiotic-resistant bacteria, the development of innovative and effective strategies that combat bacterial infections is of paramount importance. The escalating threat of bacterial infections and the rise in antibiotic resistance have spurred intensive research into novel antibacterial agents. Traditional antibiotics are ineffective against an array of pathogenic bacteria, necessitating the discovery of novel compounds with distinct modes of action. Among these, Biginelli compounds have garnered significant attention owing to their antibacterial potential;^20,21^ such compounds derived from multicomponent Biginelli reactions exhibit a broad spectrum of biological activities and antimicrobial properties. Biginelli compounds, characterized by their unique three-component syntheses, offer diverse chemical scaffolds that can be systematically modified to enhance their antibacterial efficacies. Here, we delve into the significance of Biginelli compounds as antibacterial agents and their potential to address the challenges posed by antibiotic resistance. An intriguing avenue of exploration involves the synergistic use of nanomaterials and organic compounds;^22^ this innovative approach seeks to exploit the unique properties of metal-oxide NPs along with the established antibacterial properties of Biginelli compounds to engineer advanced antibacterial systems. NPs have emerged as promising candidates in various scientific and technological domains owing to their remarkable size-dependent properties. When coupled with biocompatible gels, they can serve as versatile platforms for the controlled release of antimicrobial agents, offering a novel dimension for the development of antibacterial materials. Furthermore, among the diverse array of materials explored for their antimicrobial potentials, ZnO has emerged as a compelling candidate because it is a versatile inorganic compound that has long been recognized for its exceptional properties in various fields, including electronics, optics, and photovoltaics.^23^ Recently, its remarkable antibacterial properties have attracted considerable attention.^24,25^ ZnO exhibits potent antibacterial potential against a broad range of pathogens, thereby providing a potentially promising solution for addressing the challenges associated with antibiotic-resistant bacteria. We aimed to elucidate the unique attributes of ZnO that make it an effective antibacterial agent by considering its molecular interactions with bacterial cells. From disrupting bacterial membranes to generating reactive oxygen species (ROS), the antimicrobial mechanism associated with ZnO endows it with a broad spectrum of potential applications.^26^

The ZnO@Biginelli composite was doped into a carbomer matrix to form an antimicrobial gel. Carbopol 940 is known for its ability to form gel-like structures upon hydration; these gels are remarkably viscous and adhere strongly to surfaces, making them ideal carriers for antibacterial agents.^27^ Carbomer-based gels, with their customizable properties and potential for controlled release, are promising materials for addressing global health concerns.^28^ Through rigorous research and thorough analysis, this study aims to contribute to advancing antibacterial gels formulated with Carbopol 940, thereby offering a potential breakthrough in the fight against bacterial infections. Porous hydrophilic dressings can kill microbes faster than flat dressings. Our research aimed to elucidate the synergistic effects and enhanced antibacterial properties associated with the ZnO/Biginelli-compound/carbomer–polymer combination.

In this study, we developed a Biginelli-based ZnO-coupled carbomer gel formed by introducing a ZnO@Biginelli composite into a carbomer matrix. Biginelli compounds bearing various alkyl substituents were synthesized and characterized, after which ZnO NPs were added to each. The final gel materials obtained using these ZnO NPs and Biginelli compounds were screened for their antibacterial efficacies against Gram-positive (*Staphylococcus aureus*) and Gram-negative (*Escherichia coli*) bacteria. The ABS-G4 gel was shortlisted and coated onto wound-dressing gauze; such an antibacterial gauze was expected to be useful in medical and healthcare applications. This integration can be used to generate sophisticated systems that are potentially useful in medicine, healthcare, and other fields. This study is expected to contribute to the ongoing battle against bacterial infections.

## 2. Experimental section

### 2.1 Materials and instrumentation

All the required solvents, chemicals, and reagents required for the syntheses of the ABS-G(1–4) gels were purchased from Sigma–Aldrich, Avra Synthesis, and TCI Chemicals Private Limited and used without further purification. High-resolution mass spectra (HRMS) of the products formed was obtained using a Xevo G2-XS QTOF (WATERS) mass spectrometer. Nuclear magnetic resonance (NMR) experiments were conducted using a JEOL instrument operating at 400 and 100 MHz for ^1^H- and ^13^C-NMR, respectively, and analyzed using MestreNova software (version 14.2). Silica gel of 230–400 mesh, Merck was employed to perform column chromatography. The surface morphologies of the ABS-G4 gel, simple dressing gauze, and ABS-G4-coated dressing gauze were examined using field-emission scanning electron microscopy (FESEM) (HITACHI, SU8010 Series). The morphology of the ABS-G4 gel was also examined by atomic force microscopy (AFM, Bruker SPM Multimode 8). The synthesized gels, ZnO, and dressing gauze were subjected to powder X-ray diffraction (PXRD) in the 2–80° 2*θ* range using a RIGAKU Miniflex diffractometer with Cu Kα radiation (*λ* = 0.154 nm). An MCR-102 modular rheometer (Anton Paar, Austria) was utilized to assess the rheological parameters of the synthesized gel. Bacterial strains (*E. coli* (MTCC-119) and *S. aureus* (MTCC-740)) were purchased from IMTECH (Chandigarh, India). PerkinElmer spectrophotometer (Model-Veriton S8620G) in the range of 400–4000 cm^−1^ was utilized to obtain Fourier transform infrared (FTIR) spectra.

### 2.2 Synthesis of ZnO nanoparticles

A mixture of zinc acetate (0.1 M) and sodium hydroxide (0.1 M) was dissolved in a water/ethanol mixture and refluxed at 80 °C. The formed white precipitate was collected by filtration using Whatman filter paper and further heated at 400 °C in a muffle furnace for 3–4 h. ZnO NP formation was confirmed by PXRD.

### 2.3 General procedure for the synthesis of ABS-1–3

#### 2.3.1 Biginelli compound 1

2-Formylphenoxyacetic acid (1 g), urea (0.40 g, 1.2 equiv), ethyl acetoacetate (0.86 g, 1.2 equiv), and zinc perchlorate (catalytic amount) were dissolved in methanol (40 mL) in a round-bottom flask and refluxed for 12 h, which led to the formation of a white precipitate of the product, which was collected by filtration using Whatman filter paper and washed five-times with methanol. Thin-layer chromatography (TLC) was utilized to monitor the reaction completion. ^1^H- and ^13^C-NMR spectroscopy and HRMS confirmed that the white product was pure.

#### 2.3.2 Biginelli compound 2

Biginelli compound 1 (1 g) was dissolved in methanol (30 mL), and H_2_SO_4_ (18.4 M, five drops) was added, after which the mixture was refluxed for 8 h. An orange-yellowish product was obtained after evaporation under reduced pressure. ^1^H- and ^13^C-NMR spectroscopy and HRMS confirmed that Biginelli compound 2 was pure.

#### 2.3.3 Synthesis of ABS-B1

Biginelli compound 2 (1 g) was mixed with *n*-propylamine (0.24 mL, 1.2 equiv.) in a round bottom flask with methanol as the solvent and then refluxed at 90 °C for 6 h, which led to the formation of a white precipitate, which was collected by filtration using Whatman filter paper and washed three times with acetonitrile. ^1^H- and ^13^C-NMR spectroscopy and HRMS confirmed that the final product was pure. Similarly, Biginelli compounds ABS-B2, ABS-B3, and ABS-B4 were synthesized by reacting Biginelli compound 2 (1 g) with *n*-butylamine (0.28 mL, 1.2 equiv), *n*-octylamine (0.47 mL, 1.2 equiv), and *n*-dodecylamine (0.53 g, 1.2 equiv), respectively. All products were characterized using ^1^H- and ^13^C-NMR spectroscopy and HRMS.

#### 2.3.4 Synthesis of Biginelli-based ZnO-coupled carbomer gels (ABS-G(1–4))

ZnO (5 mg) was placed in a vessel containing distilled water (10 mL) and the mixture was ultrasonicated for 15 min. A Biginelli compound (25 mg) was dissolved in dimethyl sulfoxide (DMSO, 0.2 mL) in a separate vessel, after which the solution was added dropwise to water (10 mL) with continuous ultrasonication, with the final solution ultrasonicated for a further 15 min. Thereafter, the two solutions were mixed, and Carbopol 940 (50 mg) was added to small batches while being continuously stirred, with 50% NaOH solution used to adjust the pH to 7, resulting in the formation of carbomer-based hydrogels incorporated with ZnO NPs and Biginelli compounds. Hydrogel formation was confirmed using various characterization techniques, including SEM and AFM.

### 2.4 Rheological studies

The rheological characteristics of the ABS-G4 hydrogel were examined at 37 °C using a modular rheometer, which had a parallel plate with a 0.5 mm gap. A 400 μL hydrogel sample was prepared on the Peltier plate and incubated for 1 h. The plates were coated with liquid paraffin to reduce water evaporation during incubation. Previously reported methods were used in these rheological studies,^29,30^ which commenced with an amplitude sweep to identify the linear viscoelastic range (LVE) of the prepared hydrogel. Changes in the storage modulus were determined by subjecting the hydrogel to high (1000%) and mild (1%) strains. Furthermore, frequency sweep studies were conducted, involving the systematic variation of angular frequency within the range of 0.1 to 100 rad s^−1^ while maintaining a constant amplitude of 1%. These comprehensive rheological investigations aimed to provide a detailed understanding of the viscoelastic behavior and mechanical properties of the hydrogel under various conditions.

### 2.5 Injectability and self-healing studies

Self-healing studies were performed using a previously reported method.^30^ The self-healing properties of the ABS-G4 hydrogel were evaluated using a simple visual method, in which the gel was molded using a silicon mold into a rectangular shape, which was then cut into two equal rectangular pieces using a surgical blade. The two halves were stained with methyl orange and methylene blue dyes and placed in contact at 37 °C to observe self-healing. The injectability of ABS-G4 gel was also evaluated by taking it into a syringe and extrusion through a needle. Vial-inversion method was employed to examine the gel flow against gravity for a time interval of 10 days.

### 2.6 AFM studies

The surface topology and 2D and 3D roughness of the ABS-G4 gel were examined by atomic force microscopy (AFM, Bruker SPM Multimode 8). The developed hydrogel was taken in gel form, air dried to remove its moisture, and then subjected to AFM characterization followed by application to a silicon mold. For this analysis, the hydrogel was not coated on any surface; direct images of the hydrogel were obtained to get an idea about the surface morphology and porosity of the developed gel.

### 2.7 Preparing bacterial samples for SEM

Proper fixation, dehydration, and mounting were required to obtain high-quality SEM images. The sample containing *S. aureus* bacteria treated with the ABS-G4 gel was incubated in nutrient broth medium for one night at 37 °C, after which a 1 mL quantity of the bacterial culture was removed from the culture tube, and the cells were centrifuged to produce a cell pellet. Phosphate-buffered saline (PBS) was used to wash the cell pellet, which was fixed using a 2.5% glutaraldehyde solution in PBS (pH 7.4). The bacterial sample was dehydrated using ethanol solutions with different concentrations of 30%, 40%, 50%, 70%, 80%, and 100%. The sample was placed on a silicon wafer and air-dried at room temperature. This bacterial sample was then utilized for imaging using a scanning electron microscope.

### 2.8 Fabricating ABS-G4-coated wound-dressing gauze

A wound dressing serves as a significant barrier that shields bare skin from infectious microorganisms, thereby contributing to the efficient wound management of skin infections caused by various bacteria. Such dressing gauze offers sustained antibacterial activity over an extended period and provides long-term protection against bacterial infections and faster healing. An effective wound dressing gauze must possess non-toxic, non-adherent, and non-allergic properties. Moreover, it should exhibit remarkable antibacterial potential and be highly biocompatible.

Biginelli-compound biocompatibility was well established in the literature; at the same time, Biginelli compounds and ZnO NPs exhibited significant antibacterial properties. Consequently, the developed ABS-G4 gel was further fabricated on simple wound-dressing gauze, which involved dipping the dressing gauze into the gel.^31^ The gauze was gently removed from the gel, and the excess gel was removed using a spreader. The gauze was then air dried at room temperature and employed as antibacterial wound dressing gauze.

### 2.9 Antibacterial activity

The antibacterial activity of the synthesized gels was calculated against Gram-positive (*E. coli*) and Gram-negative bacteria (*S. aureus*). The broth microdilution method was employed to estimate the gels' minimum inhibitory concentration (MIC) values. Test tubes were filled with a standardized suspension of bacteria after the serially diluted test materials were prepared. Growth and sterility control tubes were used for every test. The tubes were incubated for the entire night at 37 °C, and their optical densities were measured at 600 nm, with minimal turbidity, providing MIC values for *E. coli* and *S. aureus* in the presence of each tested gel. Amoxicillin drug was utilized as a positive control. Further, the antibacterial efficacy of the developed ABS-G4-coated dressing gauze against *E. coli* and *S. aureus* bacteria was evaluated using the disc-diffusion method.^32^ The bacterial suspension, with a concentration of approximately 1 × 10^6^ CFU mL^−1^, was applied to the solidified agar medium using a swab and allowed to dry for 15 min. Subsequently, uncoated and ABS-G4-coated dressing gauze was placed on each disc and incubated at 37 °C for 24 h, carefully monitoring for the formation of clear inhibition zones around the gauze. The diameter of the inhibition zone in millimeters was measured to assess the antibacterial activity of the gauze.

## 3. Results and discussion

### 3.1 Syntheses

#### 3.1.1 Biginelli compound 1

2-Formylphenoxyacetic acid, urea, ethyl acetoacetate, and zinc perchlorate were dissolved in methanol and refluxed for 12 h. The reaction mixture was washed five times with methanol after being filtered. The obtained white product was pure, as confirmed by ^1^H- and ^13^C-NMR spectroscopy and HRMS. The ^1^H NMR spectrum of Biginelli compound 1 exhibited a quartet and a triplet at 3.89 and 0.97 ppm, respectively, that corresponded to the –CH_2_ and –CH_3_ groups of its alkyl chain, respectively. The singlet at 2.28 ppm corresponded to the –CH_3_ group of the dihydropyridine ring (Fig. S1†). The formation of Biginelli compound 1 was also confirmed by ^13^C-NMR spectroscopy (Fig. S2†), with its molecular ion observed at *m*/*z* 335.1241 in the high-resolution mass spectrum (Fig. S3†). These results clearly confirm a molecular formula that corresponds to the assigned structure of Biginelli compound 1.

#### 3.1.2 Biginelli compound 2

The 1 gram of Biginelli compound 1 was dissolved in 30 mL of methanol and added 4–5 drops of H_2_SO_4_ (18.4 M) to it and further refluxed the mixture for 8 h. Pure Biginelli compound 2 was obtained as an orange-yellowish product after evaporation under reduced pressure as confirmed by ^1^H- and ^13^C-NMR spectroscopy and HRMS. The ^1^H NMR spectrum (Fig. S4†) of Biginelli compound 2 exhibited a singlet at 3.81 ppm (highlighted using a yellow box) that corresponded to the three protons of the –CH_3_ group of the methyl ester moiety, confirming that the ester derivative had formed. ^13^C-NMR spectroscopy was used to confirm further that compound 2 had been prepared (Fig. S5†). Additionally, the high-resolution mass spectrum of 2 showed a molecular ion at *m*/*z* 349.1406 that confirmed the identity of Biginelli compound 2 (Fig. S6†).

#### 3.1.3 ABS-B(1–4)

Biginelli compound 2 was mixed with *n*-propylamine in methanol and heated at 90 °C for 6 h. White precipitates appeared in the reaction mixture and were filtered using the Whatman filter paper. The precipitates were further washed 2–3 times with acetonitrile. The purity of the final product (ABS-B1) was confirmed by ^1^H- and ^13^C-NMR spectroscopy and HRMS (Fig. S7–S9†). The ^1^H-NMR spectrum of ABS-B1 reveals that the peak at 3.8 ppm disappeared, and new peaks at 0.79 ppm, 1.45 ppm, 3.18 ppm, and 6.15 ppm were obtained corresponding to alkyl chain protons and –NH protons. The number of peaks obtained in the ^13^C-NMR spectrum as per the structure of ABS-B1. HRMS data further confirm its formation with a molecular ion peak at *m*/*z* 376.1887.

**Fig. 1:**
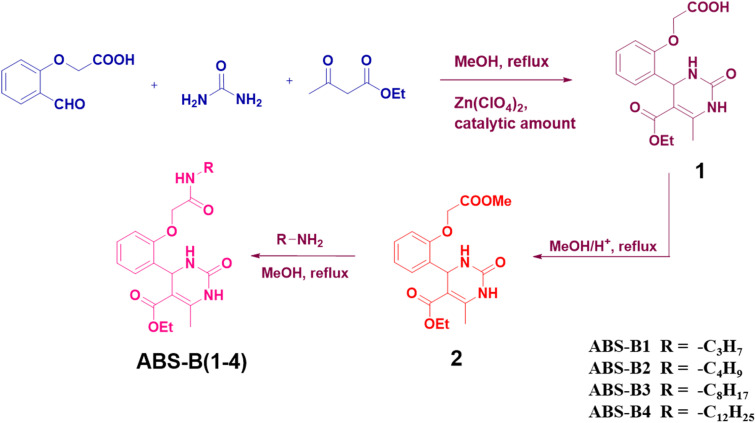
Schematic of syntheses of ABS-B(1–4).

Similarly, Biginelli compounds ABS-B(2–4) were synthesized by reacting Biginelli compound 2 with *n*-butylamine, *n*-octylamine, and *n*-dodecyl amine, respectively. All products were characterized by ^1^H-NMR (Fig. S10, S13, and S16†) and ^13^C-NMR (Fig. S11, S14, and S17†) spectroscopy, HRMS (Fig. S12, S15, and S18†) and FTIR spectroscopy (Fig. S19–S22†). The new peaks obtained upon forming these compounds from Biginelli compound 2 have been highlighted in their proton NMR spectra. A highlighted peak near 6.1 ppm corresponds to –NH– proton in each case. Other highlighted peaks in Fig. S10, S13, and S16† correspond to alkyl chain protons attached to the amide moiety. The schematic of syntheses of compounds ABS-B(1–4) has been provided in Fig. 1.

#### 3.1.4 Biginelli-based ZnO-coupled carbomer gels (ABS-G(1–4))

**Fig. 2:**
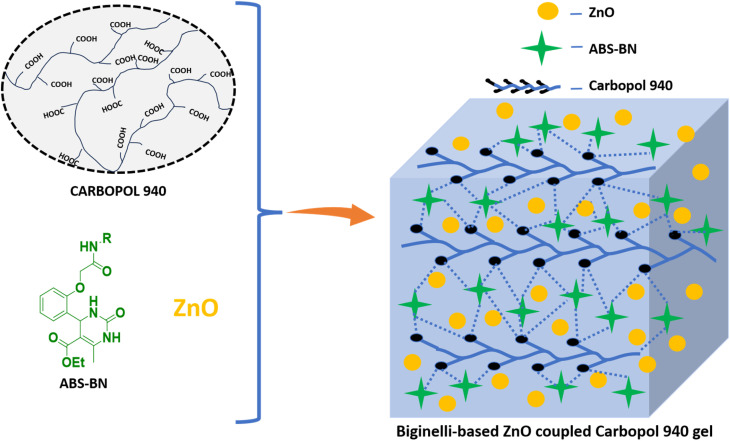
Schematic for the preparation of Biginelli-based ZnO coupled carbomer gels ABS-G(1–4).

The required Biginelli compound was dissolved in DMSO, added to ZnO in water, and then ultrasonicated. Carbopol 940 was then added to the mixture, followed by the addition of 50% aqueous NaOH solution to increase the solution pH to 7, which resulted in the formation of a carbomer-based hydrogel with incorporated ZnO NPs and the Biginelli compound (Fig. 2). The formation of the Biginelli-based ZnO-coupled carbomer gels ABS-G(1–4) was confirmed using various characterization techniques, including SEM and AFM.

### 3.2 Characterization

#### 3.2.1 PXRD spectroscopy

**Fig. 3:**
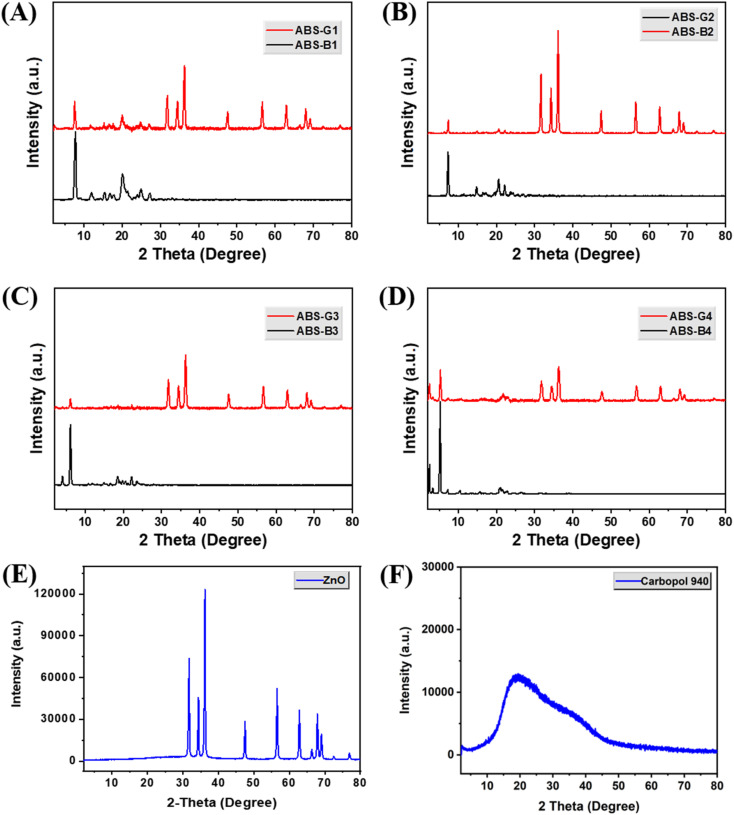
Powder X-ray diffraction (PXRD) patterns of (A)–(D) various Biginelli compounds and their corresponding ZnO-coupled carbomer gels, (E) ZnO, and (F) Carbopol 940.

The PXRD pattern recorded in the 2–80° 2*θ* range data confirmed the highly crystalline nature of the synthesized ZnO NPs (Fig. 3E), with sharp peaks observed at 2*θ* values of 31.9°, 34.5°, 36.4°, 47.6°, 56.6°, 62.9°, 66.5°, 68.0°, and 69.2° that corresponded to hexagonal wurtzite-type ZnO structure.^33^ Furthermore, the PXRD patterns of the Biginelli adducts showed characteristic peaks that corresponded to the individual molecules, as shown in Fig. 3A–D. In PXRD pattern of Biginelli compounds, prominent peaks were obtained for ABS-B1 at 7.7°,15.4°, 20.1°, 25.1°, and 27.4°; for ABS-B2 at 7.2°, 14.8°, 20.6°, and 22.1°; for ABS-B3 at 4.0°, 6.18°, 18.48°, and 22.1°; and for ABS-B4 at 2.56°, 5.34°, 7.25°, 10.5°, 15.7°, 20.9°, and 22.8°. Hence, in addition to the characteristic peaks of pristine ZnO, the patterns of the final hydrogels exhibited additional diffraction peaks that corresponded to the peaks of Biginelli compounds used to fabricate each gel material. The presence of the additional peaks in the PXRD pattern suggests that the Biginelli compounds are crystalline and likely interact with ZnO nanoparticles through different interactions including hydrogen bonding, van der Waals forces, or the formation of hybrid organic–inorganic structures.

#### 3.2.2 FESEM images and EDX spectroscopy data of the ABS-G4 gel

**Fig. 4:**
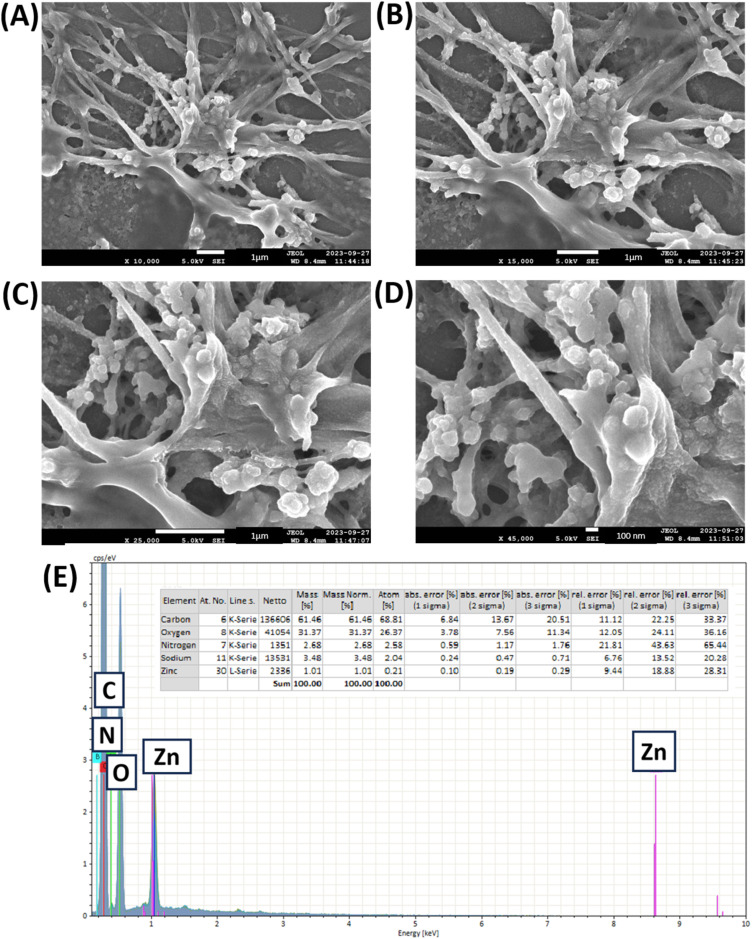
(A)–(D) Field emission scanning electron microscopy (FESEM) images of the ABS-G4 gel at various magnifications, (E) energy dispersive X-ray (EDX) spectrum of the ABS-G4 gel and corresponding data.

The Biginelli-based ZnO-coupled ABS-G4 hydrogel morphology was analyzed. FESEM images of the dried gel showed well-defined, homogeneously porous, interconnected fibrous networks, which were crucial for hydrogel stability and functionality (Fig. 4A–D).^34^ The consistency of the network structure and pore size distribution ensures that ABS-G4 hydrogel will maintain its performance across different applications, including drug delivery, wound healing, or tissue engineering. The FESEM images also revealed that the surface topology of the hydrogel contained ZnO NPs and the Biginelli compound on the surface of the carbomer polymer, which was ascribable to adhesive forces, including van der Waals interactions and hydrogen bonding. The FESEM images revealed that the hydrogel had the required porosity.^35,36^ The average pore size for the developed gel was less than 20 μm. This porosity is a crucial aspect of the functionality of the hydrogel in facilitating processes such as water absorption, retention, and controlled release of the active compounds. Further, the porous architecture enhances the stability of the hydrogel by providing structural integrity and preventing deformation during drying or utilization. The hydrogel exhibited EDX peaks that corresponded to carbon, nitrogen, oxygen, and zinc, elements confirming that the ZnO NPs and Biginelli compound were attached to the surface of the carbomer polymer (Fig. 4E).

#### 3.2.3 AFM images of the ABS-G4 gel

**Fig. 5:**
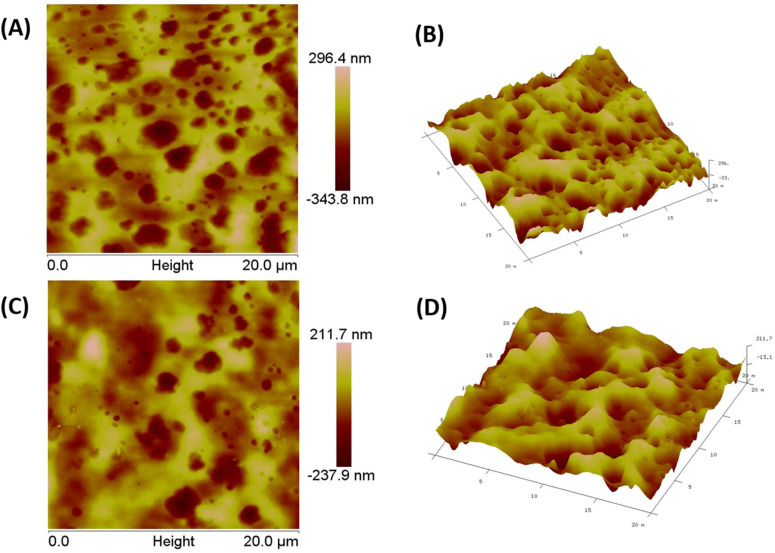
A and C represent two-dimensional atomic force microscopy data providing a picture of porosity and gel network at different portions/areas of the ABS-G4 hydrogel. Similarly, B and D represent atomic force microscopy data obtained as A and C in three-dimensional mode.

The nanoscale surface morphology and mechanical aspects of the ABS-G4 hydrogel were analyzed using AFM, which enabled high-resolution Fig. 5. Atomic force micrographs of the ABS-G4 gel in various modes imaging of the hydrogel surface and revealed the hydrogel network and its pores and identified features such as pores, fibers, or aggregates that were present on the hydrogel surface or embedded in it.^37^ The AFM images displayed in Fig. 5 revealed a gel surface that was homogeneously porous and provided comprehensive information regarding the surface morphology, including its porosity and gel network. The homogeneous porosity indicates a well-organized network of pores across the gel surface. The biomechanical properties of the hydrogel, which are important for performance in biological systems, are influenced by both the porosity and interconnected gel network. AFM results of the ABS-G4 hydrogel are in accordance with the FESEM images regarding porosity and gel network. Fig. 5A and C represent two-dimensional atomic force microscopy data providing a picture of porosity and gel network at different portions/areas of the ABS-G4 hydrogel. Similarly, Fig. 5B and D represent atomic force microscopy data obtained as Fig. 5A and C in three-dimensional mode. This provides a more comprehensive view with details about the porosity of the synthesized ABS-G4 gel.

#### 3.2.4 Rheological studies

**Fig. 6:**
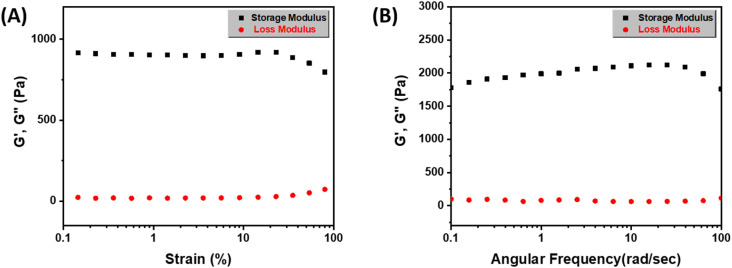
Rheologically determined viscoelastic properties of the ABS-G4 gel; storage (*G*′) and loss (*G*′′) moduli at various (A) angular frequencies, and (B) strains.

The viscoelastic properties of the ABS-G4 hydrogel were determined using rheological analysis (Fig. 6). Determining the linear viscoelastic range for hydrogels is critical because it sets the limits along which the material exhibits reliable, reproducible mechanical properties without undergoing any permanent deformation.^38^ To begin, the linear viscoelastic range was determined and used as the operating range for the subsequent rheological tests. An amplitude sweep was performed on the ABS-G4 hydrogel to assess its storage modulus and mechanical strength. The storage modulus of the hydrogel remained stable across strain values from 0.1% to 100%, confirming its mechanically resilient and viscoelastic nature (Fig. 6A). Lower loss modulus (*G*′′) value relative to storage modulus (*G*′) suggests that the ABS-G4 behaves more like a solid than a liquid and is less prone to flow under applied stress. As storage modulus (*G*′) remains largely unaffected by changes in frequency, this suggests that the hydrogel exhibits consistent elastic behavior across a wide range of deformation rates (Fig. 6B). This further confirms that ABS-G4 gel is mechanically stable and resistant to deformation.

#### 3.2.5 Self-healing capability of the hydrogel and its injectability

**Fig. 7:**
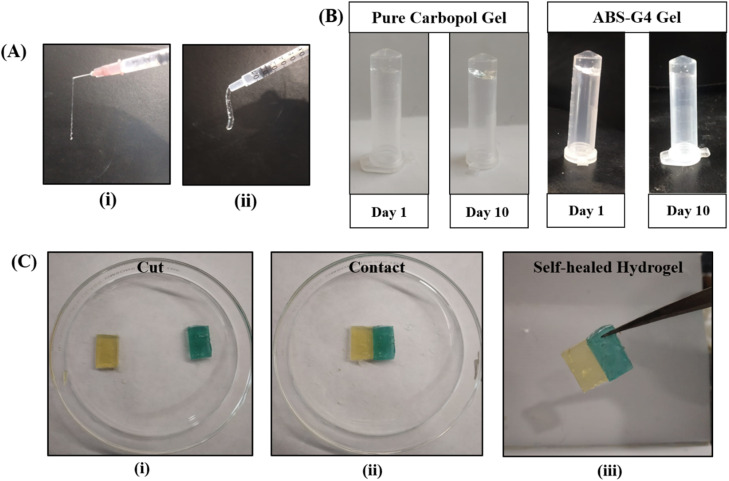
(A) Demonstrating the injectable properties of the ABS-G4 gel: (i) with and, (ii) without an18 G needle, (B) inversion testing that reveals the stability of the ABS-G4 gel under the influence of gravity, (C) demonstrating the self-healing property of the ABS-G4 gel.

The ABS-G4 hydrogel was taken into a syringe and extruded through a needle to assess its injectability, and its thixotropic properties were assessed using the obtained images (Fig. 7). The hydrogel behaved as a liquid when stress was applied, and its original gel-like properties recovered when the stress was removed. Hence, this material could be used as an injectable gel. Vial-inversion experiments were used to visually examine gel flow against gravity at different time intervals. The Biginelli-based ZnO-coupled hydrogel did not exhibit any downward flow even after 10 days of inversion (Fig. 7B), which provided further evidence for the strengths of the adhesive forces within the hydrogel structure that inhibited flow under gravity and confirmed its gel-like nature. The self-healing characteristics of the hydrogel were analysed using a visual method. The gel material was moulded in a rectangular shape by use of a silicon mold. The hydrogel was cut into two pieces after removal from the mold and these pieces were stained differently using methyl orange and methylene blue dyes. Both the stained rectangular pieces were brought together and their close contact was ensured by compression (Fig. 7C). The resultant hydrogel was then incubated in an airtight container at 37 °C for 6 h. Visual inspection of the final hydrogel revealed that it had self-healed to form a uniform gel owing to the attractive forces operating within the hydrogel. No seam was observed by microscopy, indicative of complete self-healing. This attribute offers significant advantages for hydrogels employed in various applications that include drug delivery and wound dressings. This self-healing property also significantly increases the lifespan of the hydrogel.

### 3.3 Antibacterial properties of the biginelli-based ZnO-coupled carbomer gels

The antibacterial potentials of the synthesized Biginelli-based ZnO-coupled carbomer gels were evaluated against *Escherichia coli* (gram-negative) and *Staphylococcus aureus* (gram-positive) bacteria. The colony-forming-unit (CFU) test was first used, which includes counting of viable bacterial colonies in the various cases to determine the antibacterial properties of the gel materials, with amoxicillin used as the positive control. The CFU assay revealed that these gels were capable of resisting the growth of both bacterial strains, with a minimal number of colonies present in cases that used these gels, highlighting the antibacterial potentials of the Biginelli-based ZnO-coupled carbomer gels. A broth microdilution test was used to find the minimum inhibitory concentrations (MICs) of the synthesized gels.^39,40^ The MIC had the lowest concentration of antibacterial material, which inhibited visible bacteria growth; consequently, it was used to quantify the antibacterial capabilities of the gels. Test tubes were filled with a standardized bacterial suspension along with serially diluted test materials. The optical densities of each test tube were measured at 600 nm after being incubated for an entire night at 37 °C. The minimal turbidity observed provided the MIC values for *E. coli* and *S. aureus* in the presence of each tested gel (Table 1). These findings further confirm the considerable antibacterial potential of these gels against both bacterial strains. The antibacterial activity of ZnO nanoparticles and Biginelli adducts was tested individually to understand the combination's real effect. For ZnO NPs, the minimum inhibitory concentration (MIC) values were found to be 55 μg mL^−1^ against *E. coli*, and the MIC value calculated for *S. aureus* was 40 μg mL^−1^. The results obtained are in accordance with the literature reports.^41^ The Biginelli compounds exhibited MIC values of 70–130 μg mL^−1^ against *E. coli* and *S. aureus* bacteria. The developed gel exhibited higher antibacterial efficacies, representing synergy between the ZnO NPs and Biginelli compounds. ABS-G4 exhibited the highest antibacterial efficacies among the prepared Biginelli-based ZnO-coupled carbomer gels, with MIC values of 12 ± 2 and 16 ± 2 μg mL^−1^ against *S. aureus* and *E. coli*, respectively. Hence, ABS-G4 was chosen to coat wound-dressing gauze, leading to the development of an antibacterial dressing.

**Table 1:** MICs of Biginelli-based ZnO-coupled carbomer gels against *E. coli* and *S. aureus*

Sample	Material	MIC (μg mL^−1^)	
		*E. coli* (MTCC-119)	*S. aureus* (MTCC-740)
1	ABS-G1	29 ± 2	24 ± 2
2	ABS-G2	26 ± 2	20 ± 2
3	ABS-G3	19 ± 2	15 ± 2
4	ABS-G4	16 ± 2	12 ± 2
5	Amoxicillin	04 ± 2	06 ± 2

**Fig. 8:**
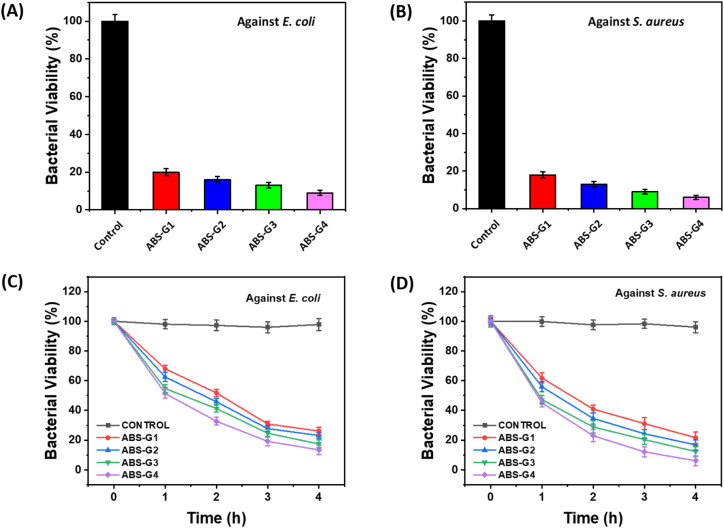
Comparative bacterial cell viabilities of (A) *E. coli* and (B) *S. aureus*, incubated with the ABS-G(1–4). Time course survival growth curves for (C) *E. coli* and (D) *S. aureus* incubated with various gels.

Fig. 8A and B shows bacterial viabilities expressed as percentages after exposure to the ABS-G(1–4) gels. The comparative antibacterial potentials of these gels against the selected bacterial strains were also analyzed over different time intervals, the results of which are shown in Fig. 8C and D. Treatment with all four gels led to remarkably fewer viable colonies of both bacterial strains after a four-hour incubation period, with ABS-G4 exhibiting the most robust antibacterial properties, demonstrating the highest growth inhibition of 85–95% for both bacterial strains. The antibacterial efficacies of the gels followed the order: ABS-G4 > ABS-G3 > ABS-G2 > ABS-G1, with differences in antibacterial effectiveness ascribable to the aliphatic chains present in the various Biginelli compounds.^42^ The aliphatic chains' hydrophobic property significantly influences Biginelli compounds' ability to cross lipid bilayers of bacterial cell membranes. Longer aliphatic chains enhance the compound's lipophilicity, allowing the molecule to penetrate the bacterial membrane and exert its antibacterial effects.^43^ Due to its excellent antibacterial potential, ABS-G4 gel is a promising candidate for various antibacterial applications.

### 3.4 Proposed mechanism for the action of ABS-G4 toward bacterial cells

**Fig. 9:**
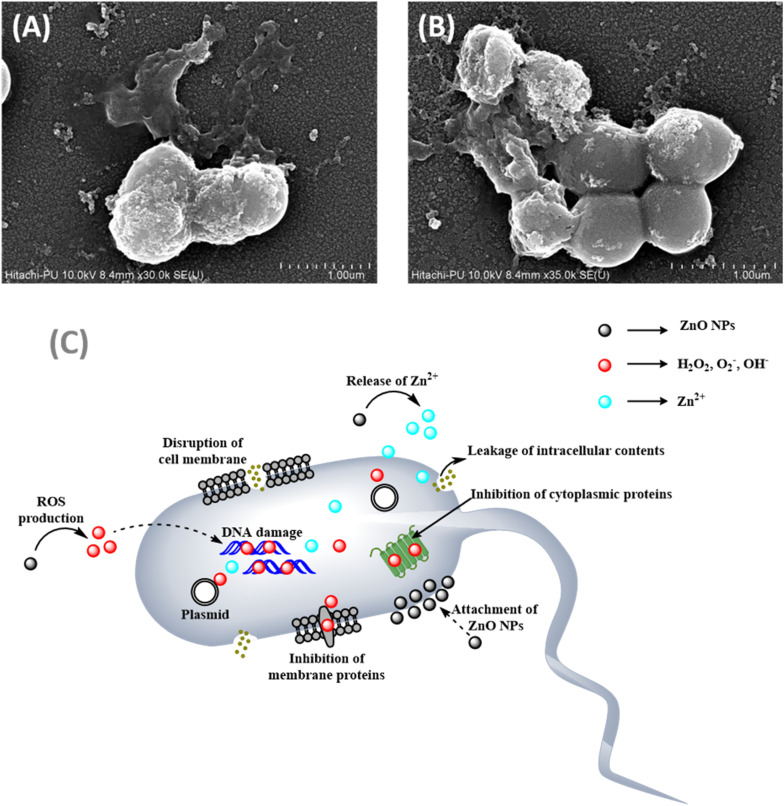
(A) and (B) Scanning electron micrographs of *S. aureus* bacteria treated with ABS-G4 gel. (C) Proposed mechanism for the antibacterial action of ZnO nanoparticles.

Elevated levels of reactive oxygen species (ROS) such as hydrogen peroxide, superoxide (O_2_˙^–^), and hydroxide (OH^−^) were reportedly produced by ZnO NPs.^44,45^ ROS oxidatively stressed bacterial cells because they were highly reactive, which damaged important cellular components including proteins, lipids, and DNA, thereby disrupting essential cellular functions and ultimately causing bacterial cell death. Furthermore, ZnO NPs released Zn^2+^ into the surrounding medium,^46^ which disrupted physiological processes within the bacteria. Excessive intracellular accumulation of Zn^2+^ could lead to cytotoxicity by interfering with cellular homeostasis. Fig. 9C shows the mechanism responsible for the antibacterial action of the ZnO NPs, which involves a combination of physical interactions, the generation of ROS, interference with cellular processes, and ion release, all of which contribute to its effectiveness against bacterial cells. The dihydropyrimidone moiety within the Biginelli compound was responsible for its antibacterial properties,^21,42^ which were observed to be affected by the length of the alkyl chain attached to the phenoxyacetate moiety. Hence, from the MIC values obtained and literature reports, the ZnO NPs and Biginelli compounds impart antibacterial potential to ABS-G4 gel through their synergistic effect. Scanning electron micrographs of *S. aureus* treated with the ABS-G4 gel are shown in Fig. 9A and B, which reveals that the bacterial cells shrank upon treatment with the ABS-G4 gel. This shrinking refers to the disruption of the cellular membrane and the inability of the cell to maintain its cellular volume, ultimately leading to bacterial cell death.

### 3.5 FESEM images of the ABS-G4-coated dressing gauze

**Fig. 10:**
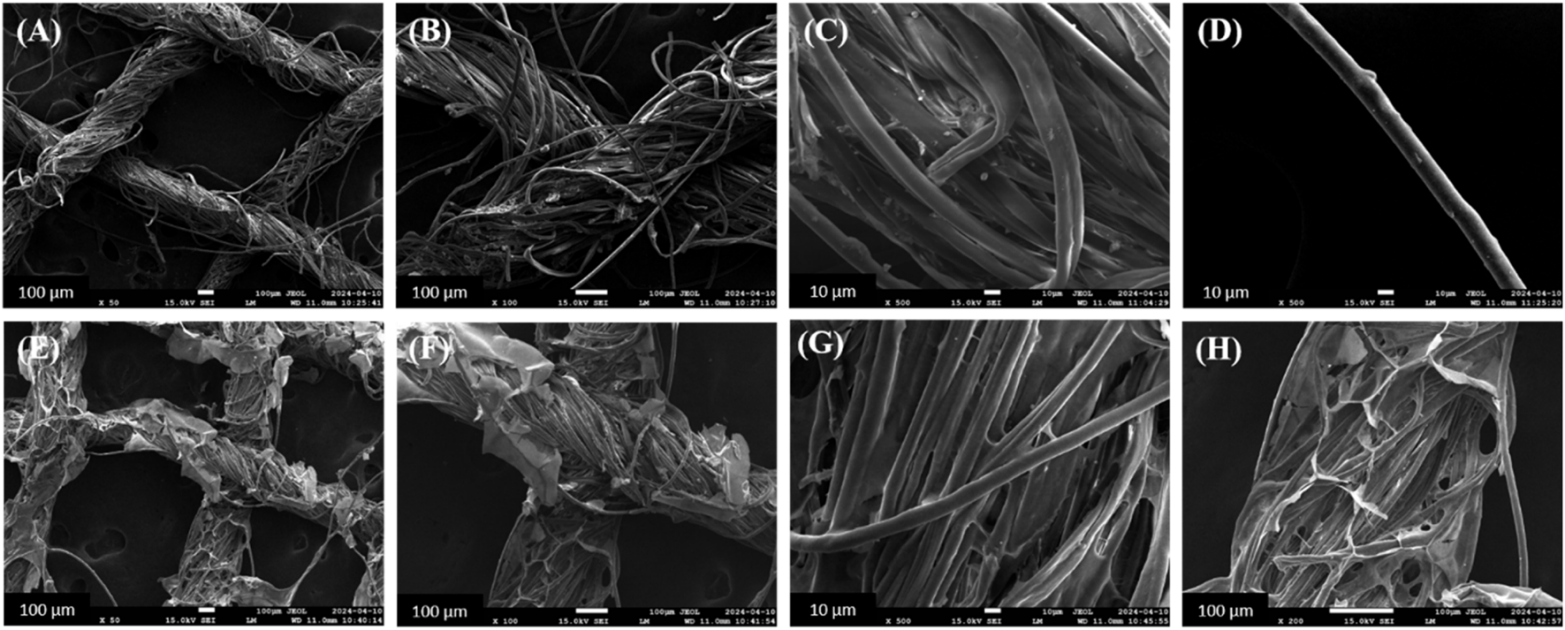
Field emission scanning electron micrographs of (A)–(D) simple dressing gauze and (E)–(H) ABS-G4-coated dressing gauze, at various magnifications.

The surface morphology of the ABS-G4 coated wound-dressing gauze was compared to that of the basic (control) dressing gauze using the scanning electron microscopy (SEM) technique. The uncoated (control) gauze exhibited a surface morphology with numerous pores, which facilitated mass transport and air diffusion through the gauze to the surrounding environment; this functionality was necessary to ensure that the wound dressing gauze was efficient and effective. The SEM images of the ABS-G4-coated dressing gauze revealed a highly porous gauze fabric, confirming that the introduction of the ABS-G4 gel did not affect the porosity of the gauze (Fig. 10) and that the gel material had been successfully embedded in the fibrous structure of the dressing gauze.

### 3.6 Antibacterial potential of the ABS-G4-coated dressing gauze

**Fig. 11:**
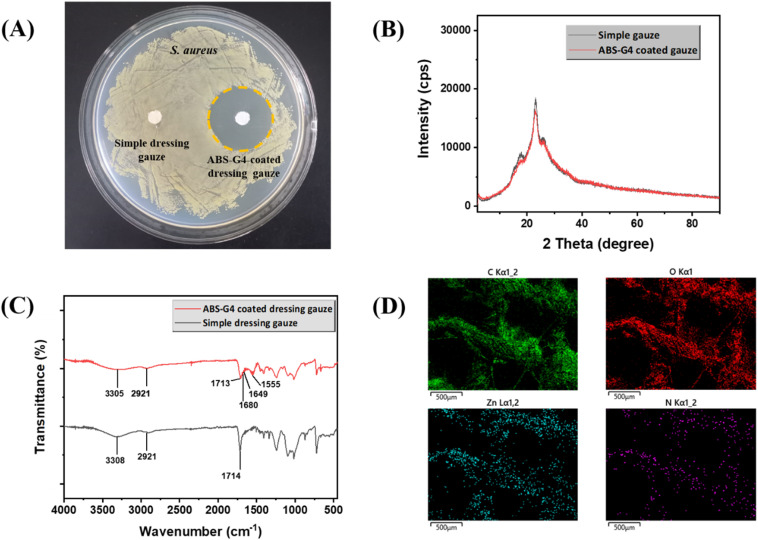
(A) Antibacterial efficacies of the ABS-G4 coated and simple (control) dressing gauze samples against *S. aureus*; (B) powder X-ray diffraction (PXRD) patterns and (C) Fourier transform infrared spectra of the two dressing gauze samples; (D) elemental maps of the ABS-G4-coated dressing gauze after 15 days after being coated.

The antibacterial potentials of both the simple and ABS-G4-coated gauze dressings were assessed against gram-positive *S. aureus* bacteria using the disc-diffusion method. Fig. 11A shows an agar plate containing the two dressing-gauze samples, which were placed in agar in a Petri dish containing medium and incubated for 24 h with an *S. aureus* suspension at 37 °C. The simple (control) dressing exhibited no significant growth inhibition. By contrast, the ABS-G4-coated dressing gauze exhibited significant inhibition against the growth of *S. aureus* bacteria with an 18 mm inhibition zone diameter. Further, we acquired PXRD patterns in the 2–80° 2*θ* range, which revealed that the two dressing-gauze samples exhibited peaks at 2*θ* values of 17.9°, 23.1°, and 25.7°, with no remarkable differences between the two observed (Fig. 11B); hence, the coating did not result in any remarkable change in the phase or amorphous nature of the dressing gauze. FTIR spectra were obtained in the range of 4000–400 cm^−1^, the results of which are shown in Fig. 11C. The simple dressing gauze exhibited a broad band in the 3600–3200 cm^−1^ range that corresponded to O–H stretching vibrations, a characteristic peak at 2921 cm^−1^ related to the stretching vibrations of C–H bond, and a noticeable peak at 1714 cm^−1^ that corresponded to >C=O stretching vibrations. The FTIR spectrum of the ABS-G4-coated wound-dressing gauze showed distinct peaks at 1649 and 1680 cm^−1^ that were indicative of the stretching vibrations of the –NH–CO–NH– moiety and the amide carbonyl (>C=O) group, along with peaks at 3305 cm^−1^ (N–H and O–H bond stretching vibrations), 2921 cm^−1^ (C–H bond stretching vibrations), and 1713 cm^−1^ (>C=O bond stretching vibrations). These FTIR spectroscopic findings confirmed that the ABS-G4 gel had been coated onto the dressing gauze. The material coated on the gauze surface remained stable even after 15 days of the natural environment's exposure, as indicated by the elemental maps of the ABS-G4-coated dressing gauze presented in Fig. 11D, which revealed that carbon, oxygen, nitrogen, and zinc elements were present on the surface of the dressing gauze, consistent with a stable and intact ABS-G4-gel coating. The ABS-G4-coated dressing gauze was economically important owing to its ease of preparation and remarkable environmental protection. Hence, this study developed a low-cost and sterile wound-dressing gauze that can be used in hospital wards, intensive care units, and other healthcare settings.

## 4. Conclusion

Biginelli-based ZnO-coupled carbomer gels with superior antibacterial activities were designed, developed, and characterized using various spectroscopic and microscopic techniques, including PXRD, FESEM, and AFM. Differently substituted Biginelli compounds were combined with ZnO NPs and doped into the carbomer matrix. Antibacterial screening revealed that the ABS-G4 gel was the most active against *E. coli* and *S. aureus* among the developed gels. MICs were determined for each gel using broth microdilution experiments, which revealed that the synthesized gels were highly antibacterially efficacious. Scanning electron microscopy provided insight into the mechanism of bactericidal action, with shrunken bacterial cell walls observed. The developed ABS-G4 gel was coated onto simple dressing gauze and characterized by FESEM, FTIR, and PXRD. The ABS-G4-coated dressing gauze significantly inhibited bacterial growth. These findings highlighted the distinct features and antibacterial potential of the Biginelli-based ZnO-coupled carbomer gel-coated dressing gauze.

## Data availability

The data that support the findings of this study are available from the first author (Bulle Shah) upon reasonable request.

## Author contributions

The manuscript was written through contributions of all authors. All authors have given approval to the final version of the manuscript. Bulle Shah – synthesis, writing – original draft, investigation. Narinder Singh – conceptualization, writing – review & editing, supervision, resources. D. O. Jang – conceptualization, validation, writing – review & editing.

## Conflicts of interest

The authors declare no competing financial interest.

## Supplementary Material

RA-015-D5RA00236B-s001
