# Supplementary material for: Development of Biginelli-based ZnO-coupled carbomer-gel-coated wound dressing gauze with enhanced antibacterial activity
Source: RSC Adv. 2025 Apr 9;15(14):11215–29. doi: 10.1039/d5ra00236b (PMC11979746; doi:10.1039/d5ra00236b)
Supplement: RA-015-D5RA00236B-s001 [file RA-015-D5RA00236B-s001.pdf]

Supplementary Information (SI) for RSC Advances.
This journal is © The Royal Society of Chemistry 2025

Supporting Information

Development of Biginelli-based ZnO-coupled carbomer-gel-coated wound dressing gauze with enhanced antibacterial activity

Bulle Shah[a], Narinder Singh[a,*], Doo Ok Jang[b,*]

[a]*Department of Chemistry, Indian Institute of Technology Ropar, Rupnagar, Punjab140001, India*

[b]*Department of Chemistry, Yonsei University, Wonju 26493, Republic of Korea*

*Corresponding author e-mail: nsingh@iitrpr.ac.in (N. Singh), dojang@yonsei.ac.kr (D. O. Jang)

Table of contents:

Figure S1: ^{1}H NMR spectrum of compound **1**.

Figure S2: ^{13}C NMR spectrum of compound **1**.

Figure S3: High resolution mass spectrum of compound **1**.

Figure S4: ^{1}H NMR spectrum of compound **2**.

Figure S5: ^{13}C NMR spectrum of compound **2**.

Figure S6: High resolution mass spectrum of compound **2**.

Figure S7: ^{1}H NMR spectrum of compound **ABS-B1**.

Figure S8: ^{13}C NMR spectrum of compound **ABS-B1**.

Figure S9: High resolution mass spectrum of compound **ABS-B1**.

Figure S10: ^{1}H NMR spectrum of compound **ABS-B2**.

Figure S11: ^{13}C NMR spectrum of compound **ABS-B2**.

Figure S12: High resolution mass spectrum of compound **ABS-B2**.

Figure S13: ^{1}H NMR spectrum of compound **ABS-B3**.

Figure S14: ^{13}C NMR spectrum of compound **ABS-B3**.

Figure S15: High resolution mass spectrum of compound **ABS-B3**.

Figure S16: ^{1}H NMR spectrum of compound **ABS-B4**.

Figure S17: ^{13}C NMR spectrum of compound **ABS-B4**.

Figure S18: High resolution mass spectrum of compound **ABS-B4**.

Figure S19: FT-IR spectrum of compound **ABS-B1**.

Figure S20: FT-IR spectrum of compound **ABS-B2**.

Figure S21: FT-IR spectrum of compound **ABS-B3**.

Figure S22: FT-IR spectrum of compound **ABS-B4**.

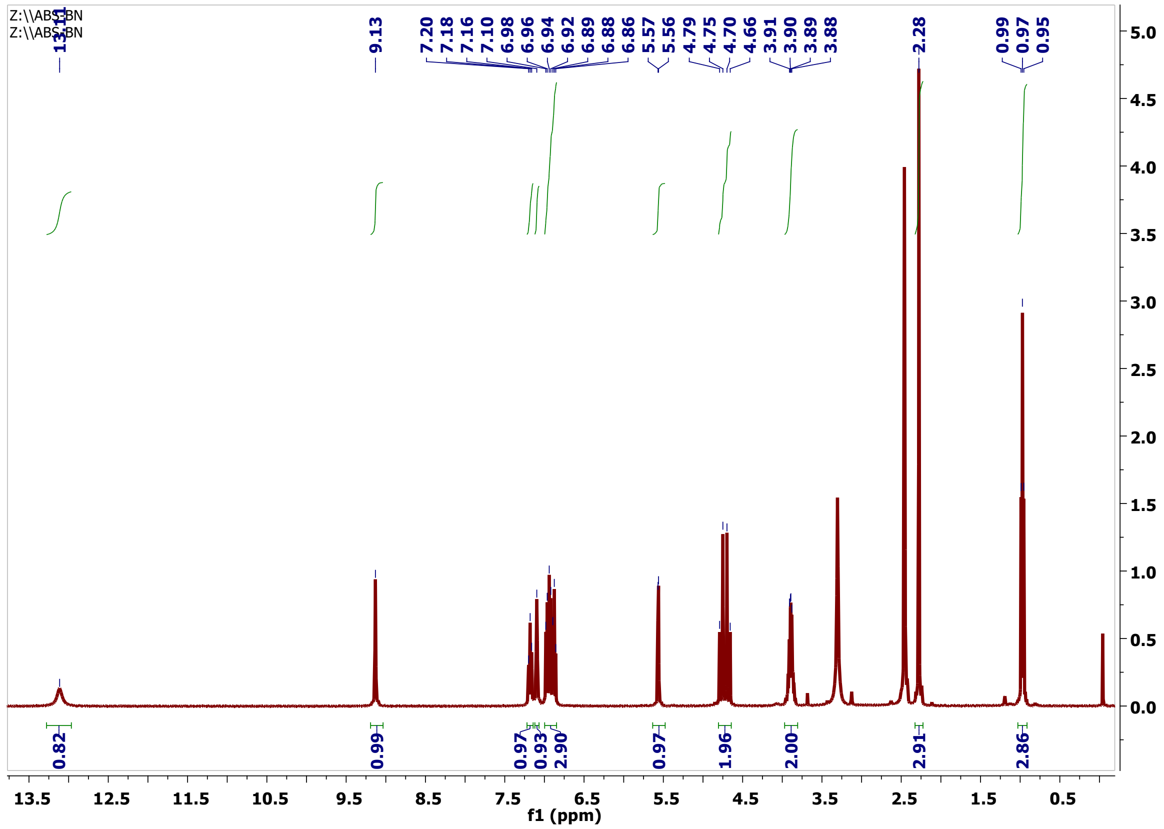

Figure S1: ^{1}H NMR spectrum of compound **1**.

^{1}H NMR (400 MHz, DMSO-D6) δ 13.11 (s, 1H, -OH), 9.13 (s, 1H, -NH), 7.18 (t, *J* = 8 Hz, 1H, -NH), 7.09 (s, 1H, -NH), 6.86 - 6.98 (m, 3H, Ar), 5.57 (d, *J* = 2.4 Hz, 1H), 4.72 (q, *J* = 20.4 Hz, 2H), 3.89 (q, *J* = 4.8 Hz, 2H), 2.28 (s, 3H), 0.97 (t, *J* = 7.2 Hz, 3H).

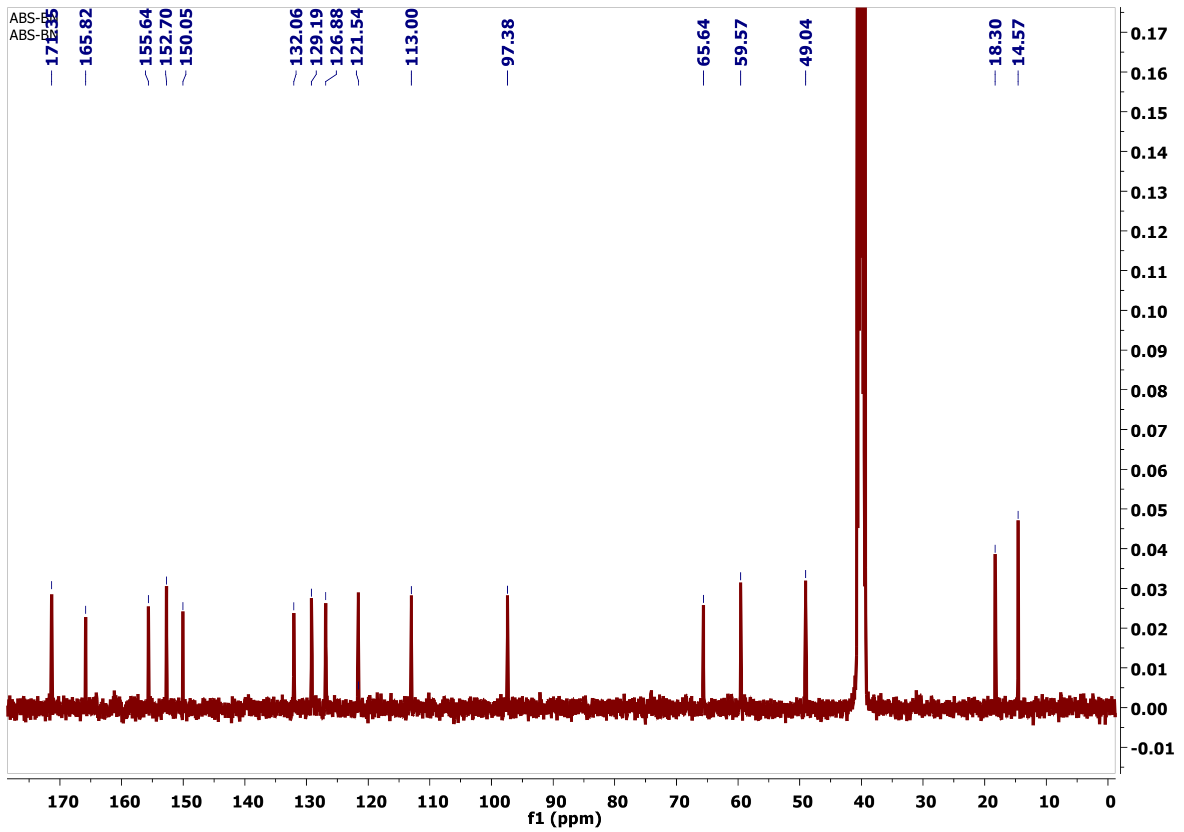

Figure S2: ^{13}C NMR spectrum of compound **1.**

^{13}C NMR (100 MHz, DMSO-D6) δ 171.35, 165.82, 155.64, 152.70, 150.05, 132.06, 129.19, 126.88, 121.54, 113.00, 97.38, 65.64, 59.57, 49.04, 18.30, 14.57.

Elemental Composition Report **Page 1**

Single Mass Analysis
Tolerance = 5.0 mDa / DBE: min = -1.5, max = 50.0
Element prediction: Off
Number of isotope peaks used for i-FIT = 3

Monoisotopic Mass, Even Electron Ions
28 formula(e) evaluated with 1 results within limits (up to 50 closest results for each mass)
Elements Used:
C: 1-17 H: 1-100 N: 1-3 O: 1-6

280623_ABS_BN 42 (0.446) IITRPR XEVO G2-XS QTOF
Test Name :
280623_ABS_BN
1: TOF MS ES+
1.52e+006

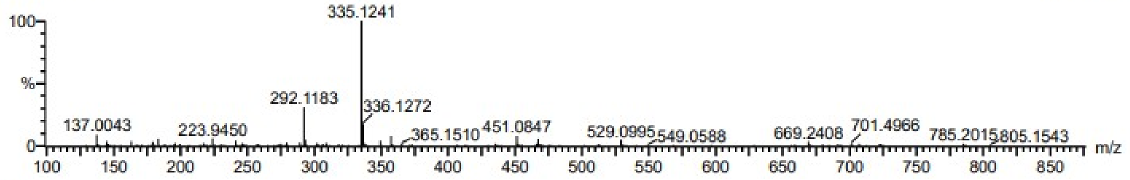

Minimum:				-1.5				
Maximum:		5.0	20.0	50.0				
Mass	Calc. Mass	mDa	PPM	DBE	i-FIT	Norm	Conf(%)	Formula
335.1241	335.1243	-0.2	-0.6	8.5	876.6	n/a	n/a	C16 H19 N2 O6

Figure S3: High resolution mass spectrum of compound **1**.

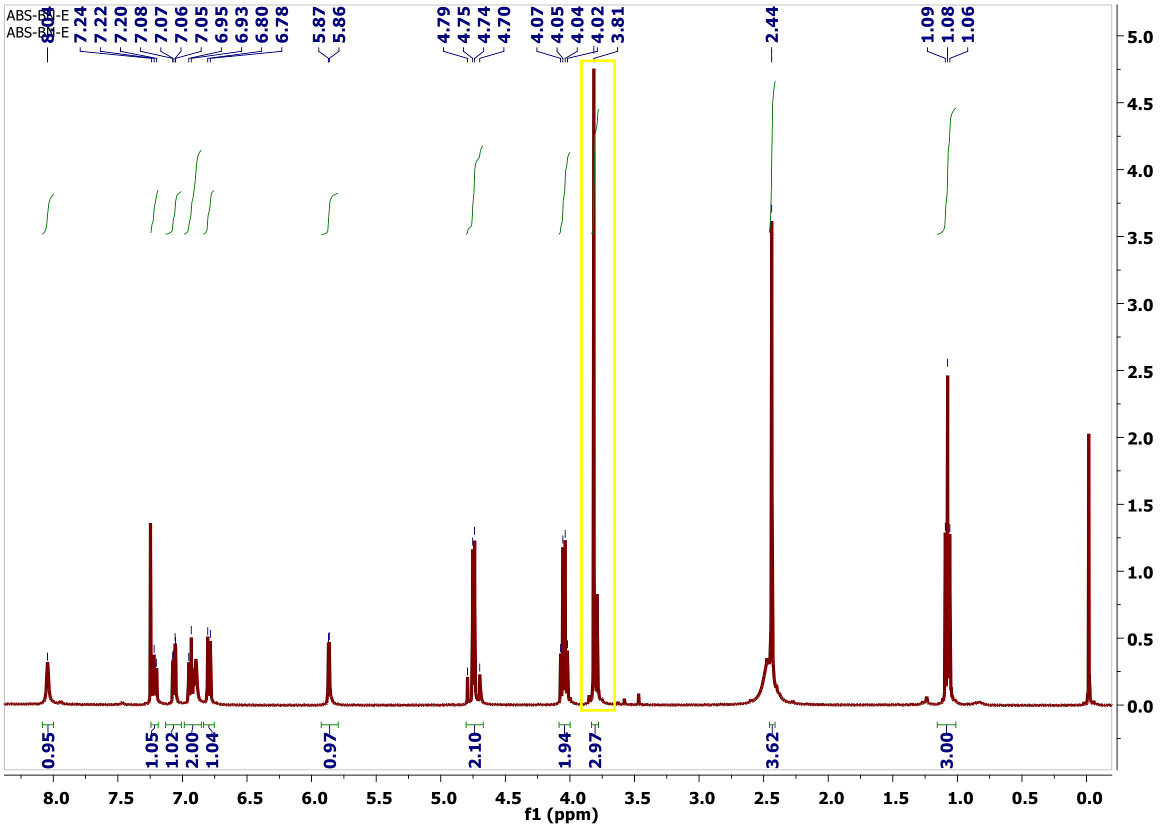

Figure S4: ^{1}H NMR spectrum of compound **2**.

^{1}H NMR (400 MHz, CHLOROFORM-D) δ 8.04 (s, 1H, -NH), 7.22 (t, J = 8 Hz, 1H, -NH), 7.05 - 7.08 (dd, J = 7.6, 1.6 Hz, 1H), 6.90 – 6.95 (m, 2H, Ar), 6.79 (d, J = 8 Hz, 1H), 5.87 (d, J = 2.8 Hz, 1H), 4.75 (q, J = 16 Hz, 2H), 4.05 (q, J = 7.2 Hz, 2H), 3.81 (s, 3H), 2.44 (s, 3H), 1.08 (t, J = 7.2 Hz, 3H).

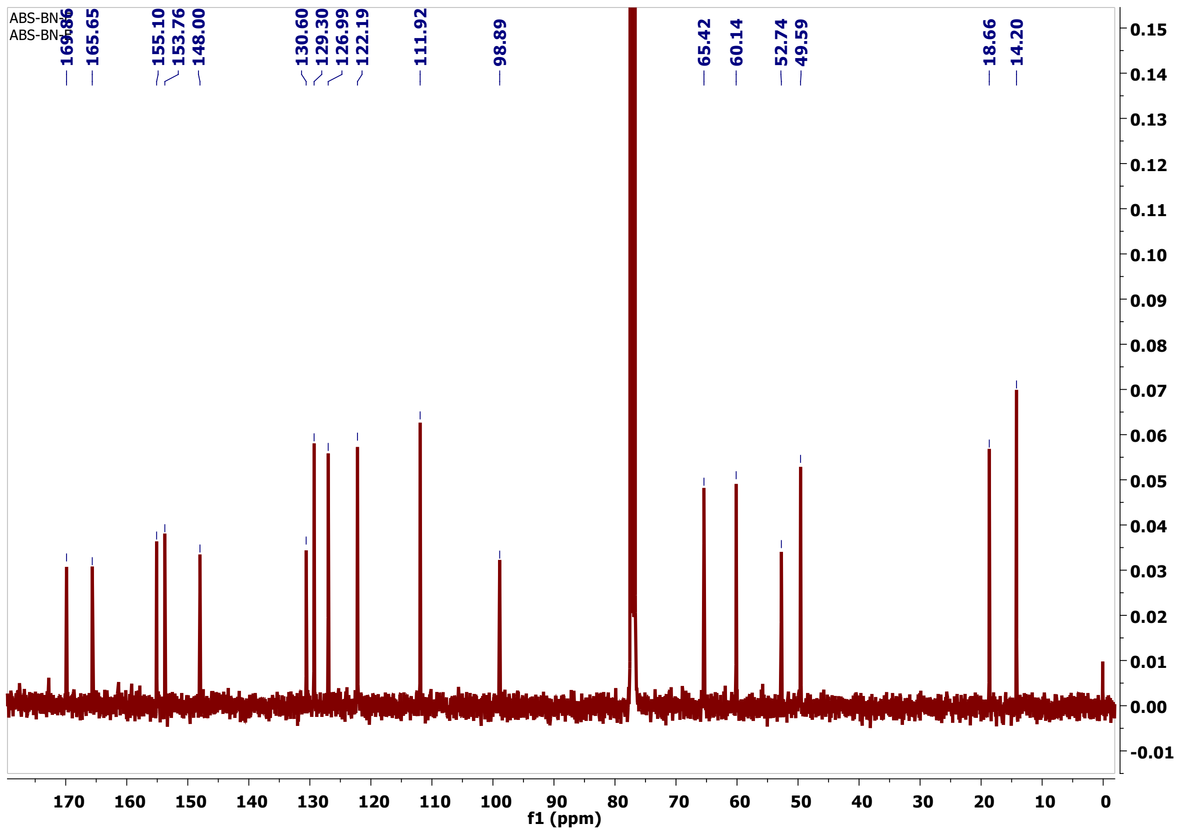

Figure S5: ^{13}C NMR spectrum of compound **2**.

^{13}C NMR (100 MHz, CHLOROFORM-D) δ 169.86, 165.65, 155.10, 153.76, 148.00, 130.60, 129.30, 126.99, 122.19, 111.92, 98.89, 65.42, 60.14, 52.74, 49.59, 18.66, 14.20.

Elemental Composition Report **Page 1**

Single Mass Analysis
Tolerance = 5.0 mDa / DBE: min = -1.5, max = 50.0
Element prediction: Off
Number of isotope peaks used for i-FIT = 3

Monoisotopic Mass, Even Electron Ions
23 formula(e) evaluated with 1 results within limits (up to 50 closest results for each mass)
Elements Used:
C: 1-17 H: 1-100 N: 1-3 O: 1-6

280623_ABS_BN_E 40 (0.417) IITRPR XEVO G2-XS QTOF
Test Name : 280623_ABS_BN_E
1: TOF MS ES+
9.91e+006

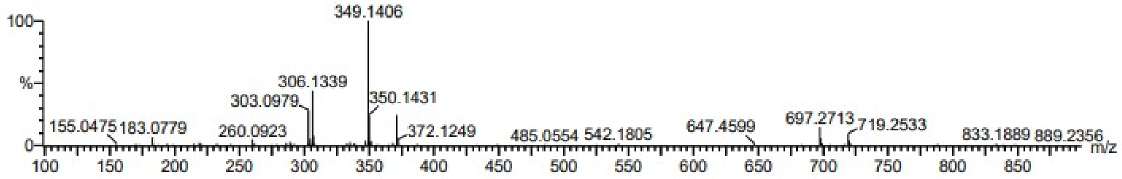

Minimum:				-1.5				
Maximum:		5.0	20.0	50.0				
Mass	Calc. Mass	mDa	PPM	DBE	i-FIT	Norm	Conf(%)	Formula
349.1406	349.1400	0.6	1.7	8.5	976.7	n/a	n/a	C17 H21 N2 O6

Figure S6: High resolution mass spectrum of compound **2**.

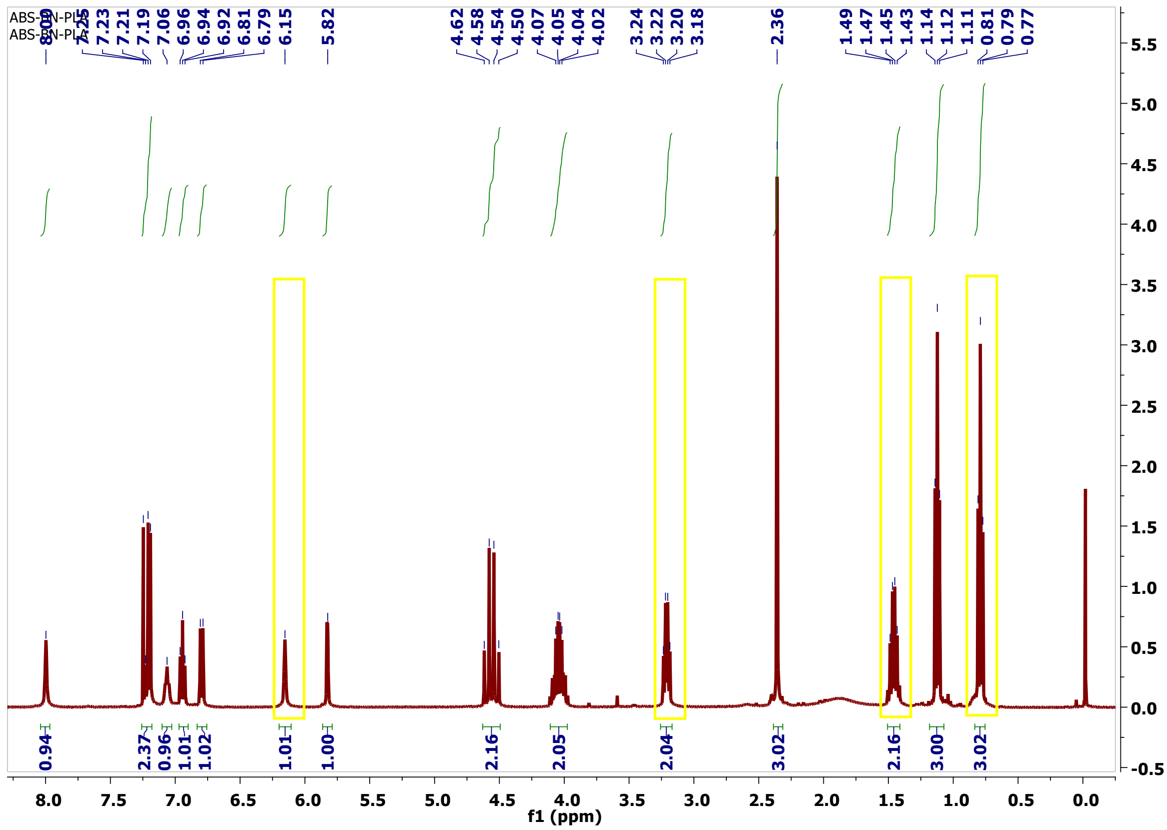

Figure S7: ^{1}H NMR spectrum of compound **ABS-B1**.

^{1}H NMR (400 MHz, CHLOROFORM-D) δ 8.00 (s, 1H, -NH), 7.25 – 7.19 (m, 2H, Ar), 7.06 (t, *J* = 5.2 Hz, 1H, -NH), 6.94 (t, *J* = 7.6 Hz, 1H, -Ar), 6.80 (d, *J* = 8 Hz, 1H, Ar), 6.15 (s, 1H, -NH), 5.82 (d, *J* = 2.4 Hz ,1H), 4.62 – 4.50 (q, *J* = 14.8 Hz, 2H), 4.07 – 4.02 (m, 2H), 3.24 – 3.18 (q, 2H), 2.36 (s, 3H), 1.49 – 1.43 (m, 2H), 1.12 (t, *J* = 7.2 Hz, 3H), 0.79 (t, *J* = 7.6 Hz, 3H).

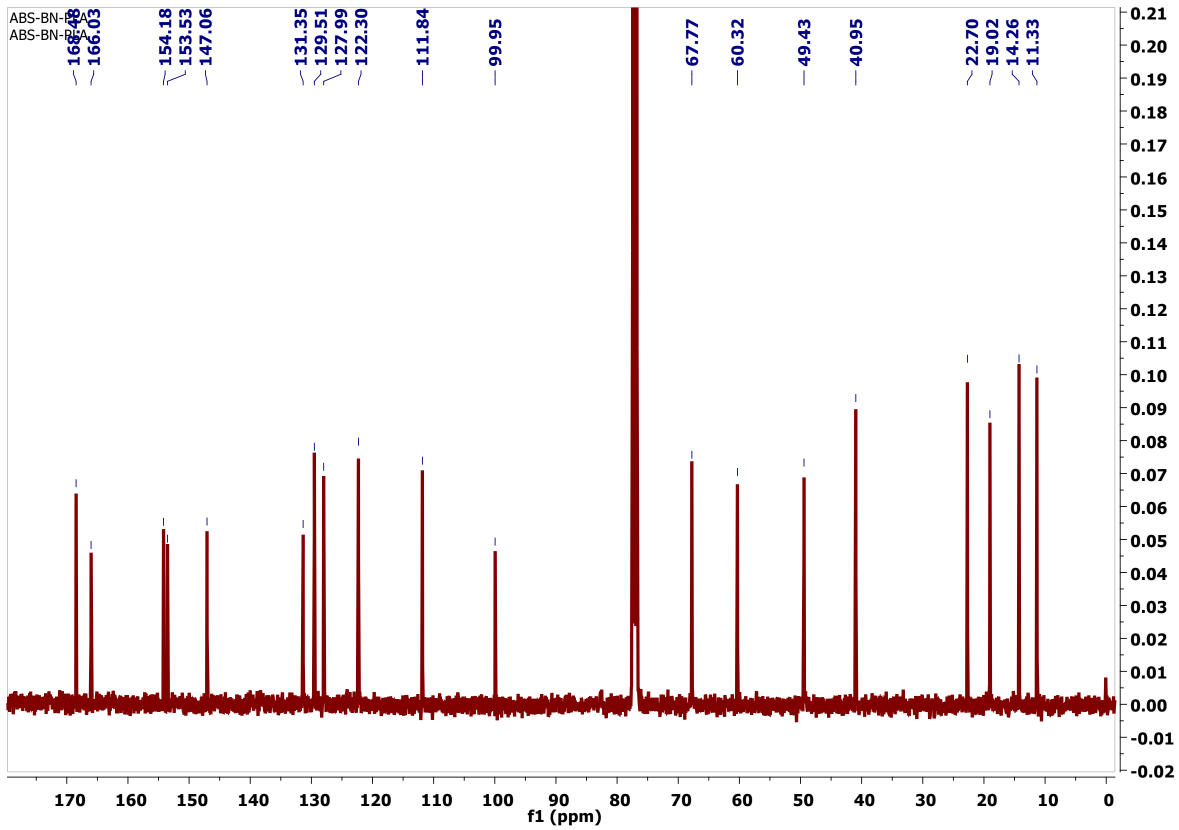

Figure S8: ^{13}C NMR spectrum of compound **ABS-B1**.

^{13}C NMR (100 MHz, CHLOROFORM-D) δ 168.48, 166.03, 154.18, 153.53, 147.06, 131.35, 129.51, 127.99, 122.30, 111.84, 99.95, 67.77, 60.32, 49.43, 40.95, 22.70, 19.02, 14.26, 11.33.

Elemental Composition Report **Page 1**

Single Mass Analysis
Tolerance = 5.0 mDa / DBE: min = -1.5, max = 50.0
Element prediction: Off
Number of isotope peaks used for i-FIT = 3

Monoisotopic Mass, Even Electron Ions
64 formula(e) evaluated with 1 results within limits (up to 50 closest results for each mass)
Elements Used:
C: 0-19 H: 0-50 N: 0-3 O: 0-5 P: 0-1

020223_ABS_BN_PLA 25 (0.277) IITRPR XEVO G2-XS QTOF
Test Name : 020223_ABS_BN_PLA
1: TOF MS ES+ 1.07e+007

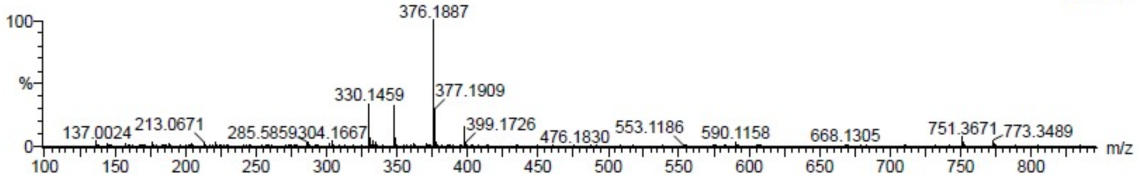

Minimum:				-1.5				
Maximum:		5.0	10.0	50.0				
Mass	Calc. Mass	mDa	PPM	DBE	i-FIT	Norm	Conf(%)	Formula
376.1887	376.1872	1.5	4.0	8.5	1020.6	n/a	n/a	C19 H26 N3 O5

Figure S9: High resolution mass spectrum of compound **ABS-B1**.

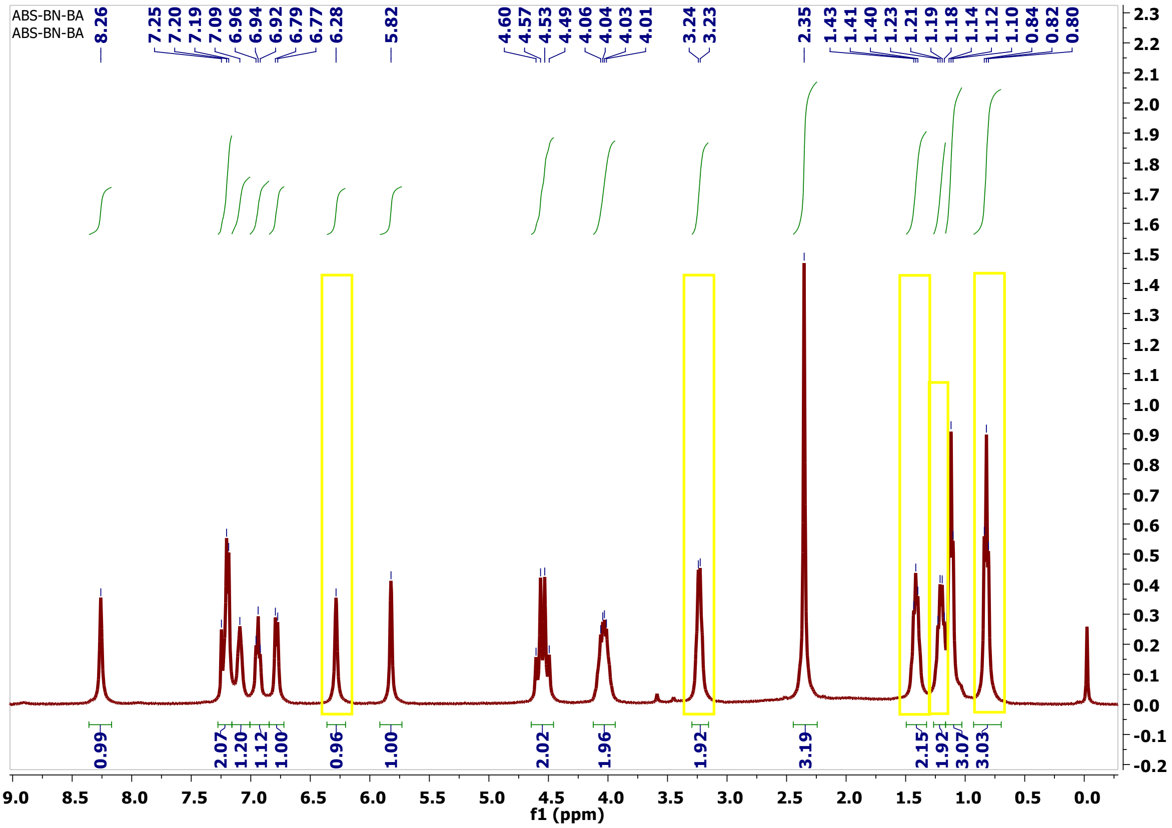

Figure S10: ^{1}H NMR spectrum of compound **ABS-B2**.

^{1}H NMR (400 MHz, CHLOROFORM-D) δ 8.26 (s, 1H, -NH), 7.20 (m, 2H, Ar), 7.09 (s, 1H, Ar), 6.94 (t, *J* = 7.2 Hz ,1H, -NH), 6.78 (d, *J* = 8 Hz, 1H, Ar), 6.28 (s, 1H, -NH), 5.82 (s, 1H), 4.60 – 4.49 (q, *J* = 14 Hz, 2H), 4.06 – 4.01 (m, 2H), 3.24 (m, 2H), 2.35 (s, 3H), 1.43 – 1.40 (m, 2H), 1.23 – 1.18 (m, 2H), 1.12 (t, *J* = 6.8 Hz, 3H), 0.82 (t, *J* = 6.8 Hz, 3H)

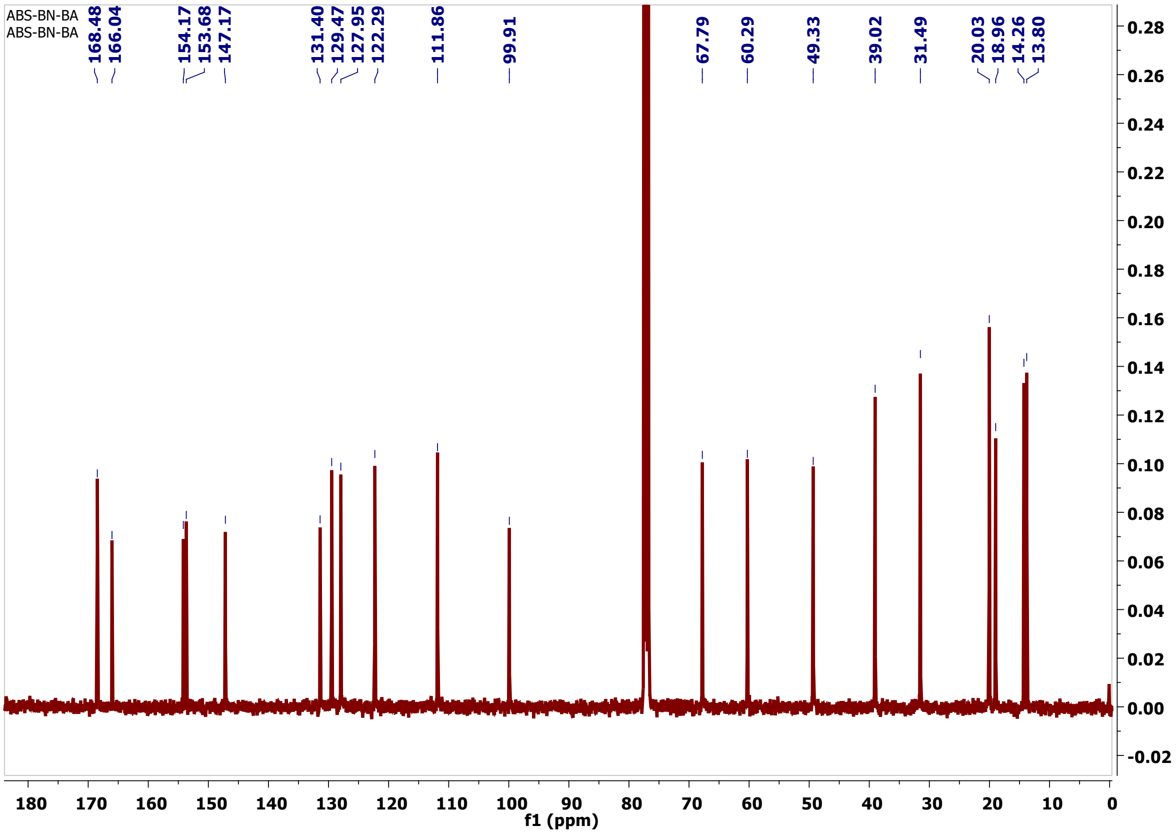

Figure S11: ^{13}C NMR spectrum of compound **ABS-B2**.

^{13}C NMR (100 MHz, CHLOROFORM-D) δ 168.48, 166.04, 154.17, 153.68, 147.17, 131.40, 129.47, 127.95, 122.29, 111.86, 99.91, 67.79, 60.29, 49.33, 39.02, 31.49, 20.03, 18.96, 14.26, 13.80.

Elemental Composition Report **Page 1**

Single Mass Analysis
Tolerance = 5.0 mDa / DBE: min = -1.5, max = 50.0
Element prediction: Off
Number of isotope peaks used for i-FIT = 3

Monoisotopic Mass, Even Electron Ions
119 formula(e) evaluated with 1 results within limits (up to 50 closest results for each mass)
Elements Used:
C: 0-24 H: 0-50 N: 0-3 O: 0-5 P: 0-1

020223_ABS_BN_BA 24 (0.257) IITRPR XEVO G2-XS QTOF
Test Name : 020223_ABS_BN_BA
1: TOF MS ES+

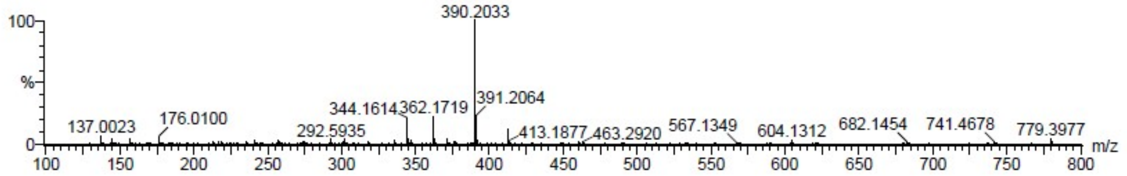

Minimum:				-1.5				
Maximum:		5.0	10.0	50.0				
Mass	Calc. Mass	mDa	PPM	DBE	i-FIT	Norm	Conf(%)	Formula
390.2033	390.2029	0.4	1.0	8.5	963.3	n/a	n/a	C20 H28 N3 O5

Figure S12: High resolution mass spectrum of compound **ABS-B2**.

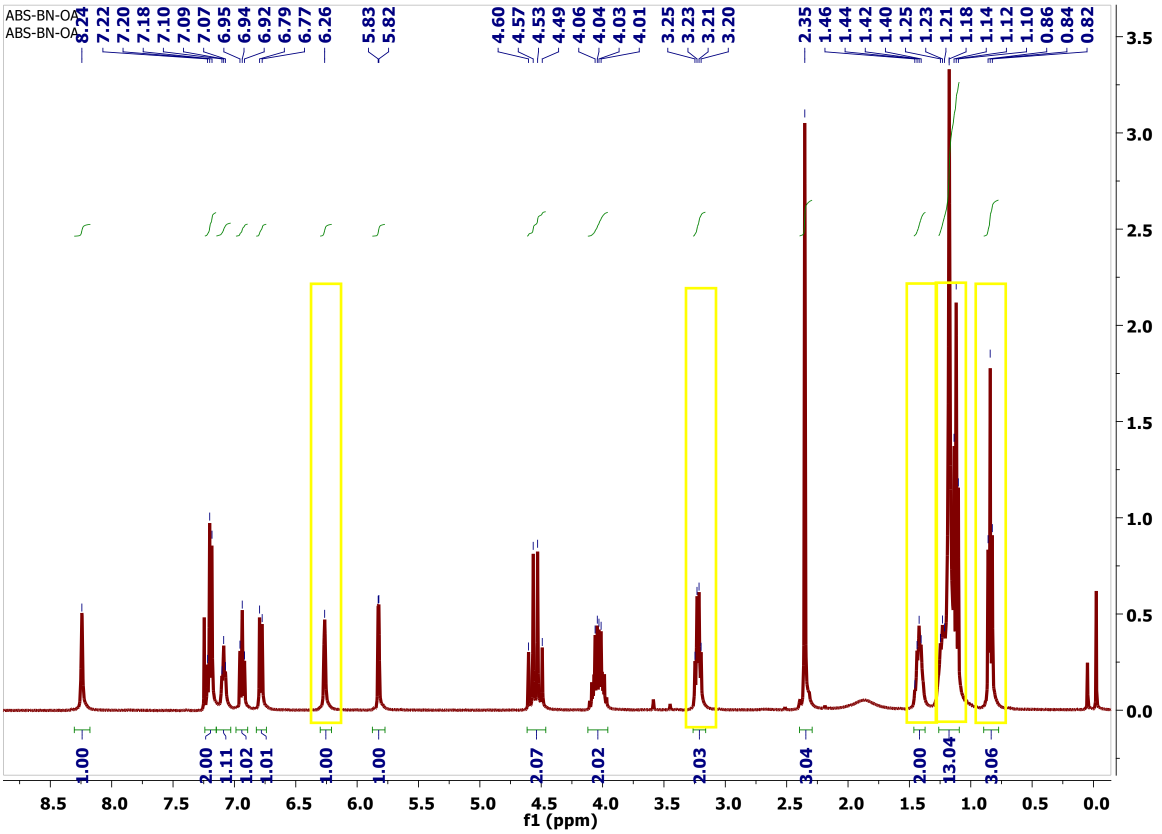

Figure S13: ^{1}H NMR spectrum of compound **ABS-B3**.

^{1}H NMR (400 MHz, CHLOROFORM-D) δ 8.24 (s, 1H, -NH), 7.20 (m, 2H, Ar), 7.09 (t, *J* = 5.6 Hz, 1H, -NH), 6.94 (t, *J* = 7.6 Hz, 1H, Ar), 6.78 (d, *J* = 8.4 Hz, 1H, Ar), 6.26 (s, 1H, -NH), 5.82 (d, *J* = 2 Hz, 1H), 4.60 – 4.49 (q, *J* = 15.2 Hz, 2H), 4.06 – 4.01 (m, 2H), 3.25 – 3.20 (q, *J* = 6.8 Hz, 2H), 2.35 (s, 3H), 1.46 – 1.40 (m, 2H), 1.25 – 1.10 (m, 13H), 0.84 (t, *J* = 7.2 Hz, 3H).

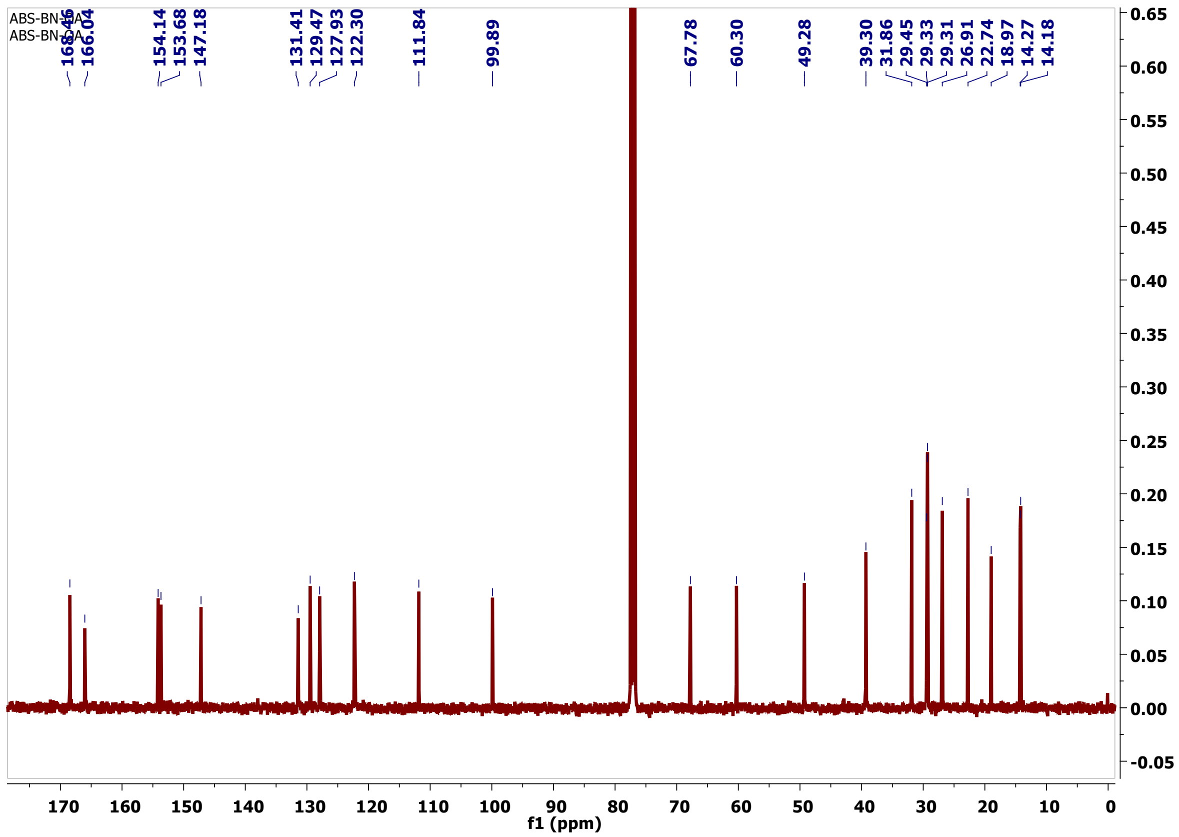

Figure S14: ^{13}C NMR spectrum of compound **ABS-B3**.

^{13}C NMR (100 MHz, CHLOROFORM-D) δ 168.46, 166.04, 154.14, 153.68, 147.18, 131.41, 129.47, 127.93, 122.30, 111.84, 99.89, 67.78, 60.30, 49.28, 39.30, 31.86, 29.45, 29.33, 29.31, 26.91, 22.74, 18.97, 14.27, 14.18.

Elemental Composition Report

Page 1

Single Mass Analysis

Tolerance = 5.0 mDa / DBE: min = -1.5, max = 50.0
Element prediction: Off
Number of isotope peaks used for i-FIT = 3

Monoisotopic Mass, Even Electron Ions
56 formula(e) evaluated with 1 results within limits (up to 50 closest results for each mass)
Elements Used:
C: 0-24 H: 0-50 N: 0-3 O: 0-5 P: 0-1

020223_ABS_BN_DA 23 (0.248) IITRPR XEVO G2-XS QTOF
Test Name : 020223_ABS_BN_DA
1: TOF MS ES+ 1.27e+007

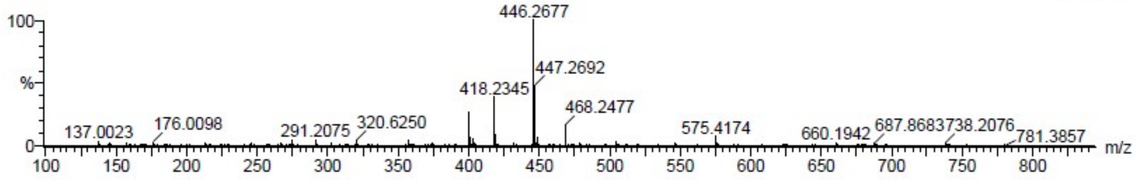

Minimum:				-1.5				
Maximum:		5.0	10.0	50.0				
Mass	Calc. Mass	mDa	PPM	DBE	i-FIT	Norm	Conf(%)	Formula
446.2677	446.2655	2.2	4.9	8.5	942.8	n/a	n/a	C24 H36 N3 O5

Figure S15: High resolution mass spectrum of compound **ABS-B3**.

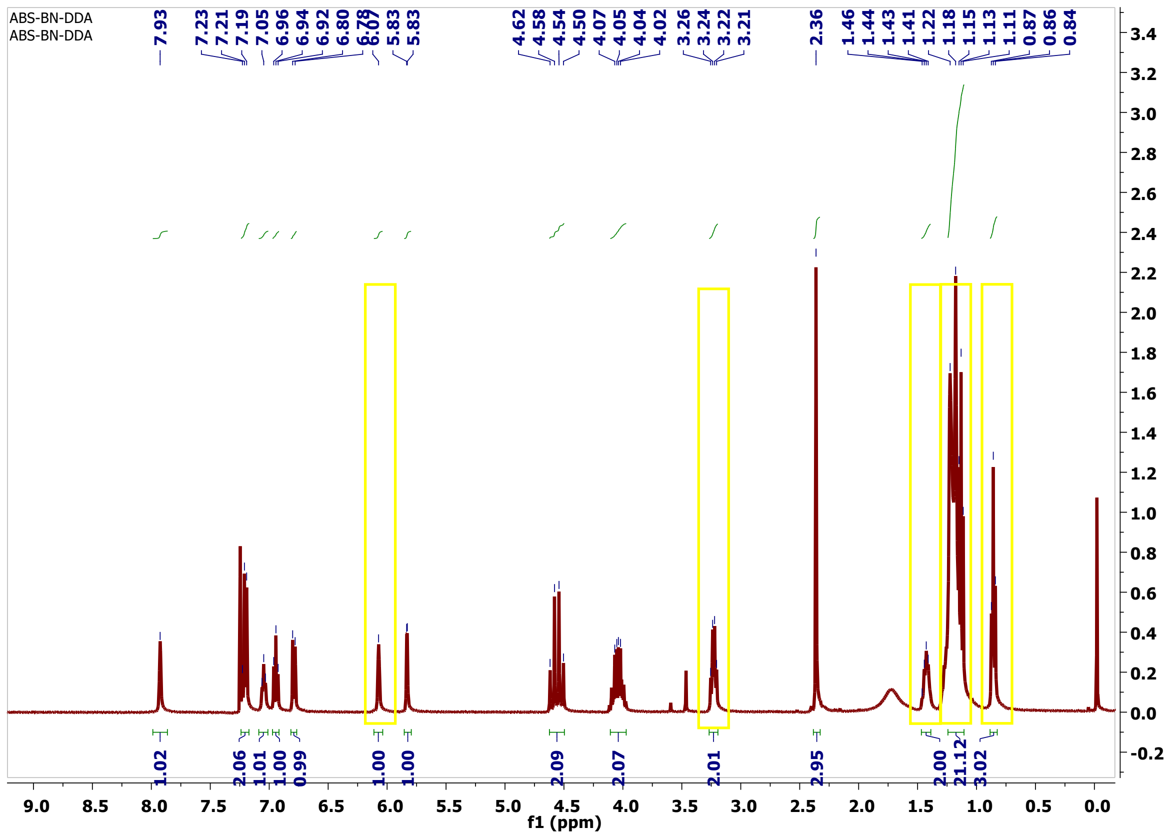

Figure S16: ^{1}H NMR spectrum of compound **ABS-B4**.

^{1}H NMR (400 MHz, CHLOROFORM-D) δ 7.93 (s, 1H, -NH), 7.21 (m, 2H, Ar), 7.05 (t, *J* = 5.2 Hz, 1H, -NH), 6.94 (t, *J* = 7.2 Hz, 1H, Ar), 6.78 (d, *J* = 8.4 Hz, 1H, Ar), 6.07 (s, 1H, -NH), 5.83 (d, *J* = 2.4 Hz, 1H), 4.62 – 4.50 (q, *J* = 15.2 Hz, 2H), 4.07 – 4.02 (m, 2H), 3.26 – 3.21 (q, *J* = 6.8 Hz, 2H), 2.36 (s, 3H), 1.46 – 1.41 (m, 2H), 1.22 – 1.11 (m, 21H), 0.86 (t, *J* = 7.2 Hz, 3H).

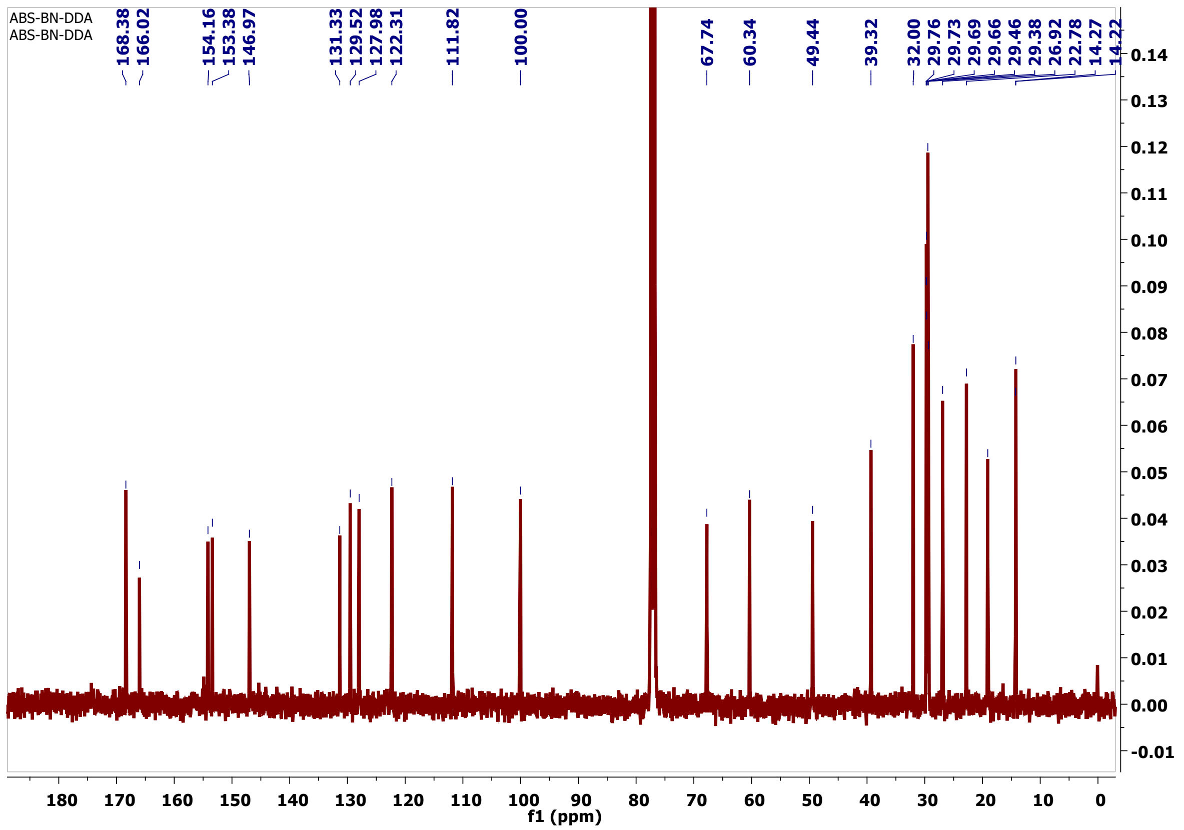

Figure S17: ^{13}C NMR spectrum of compound **ABS-B4**.

^{13}C NMR (100 MHz, CHLOROFORM-D) δ 168.38, 166.02, 154.16, 153.38, 146.97, 131.33, 129.52, 127.98, 122.31, 111.82, 100.00, 67.74, 60.34, 49.44, 39.32, 32.00, 29.76, 29.73, 29.69, 29.66, 29.46, 29.38, 26.92, 22.78, 19.09, 14.27, 14.22.

Elemental Composition Report Page 1

Single Mass Analysis
Tolerance = 5.0 mDa / DBE: min = -1.5, max = 50.0
Element prediction: Off
Number of isotope peaks used for i-FIT = 3

Monoisotopic Mass, Even Electron Ions
47 formula(e) evaluated with 1 results within limits (up to 50 closest results for each mass)
Elements Used:
C: 0-28 H: 0-50 N: 0-3 O: 0-5 P: 0-1

020223_ABS_BN_DDA 23 (0.248) IITRPR XEVO G2-XS QTOF
Test Name :
1: TOF MS ES+ 020223_ABS_BN_DDA

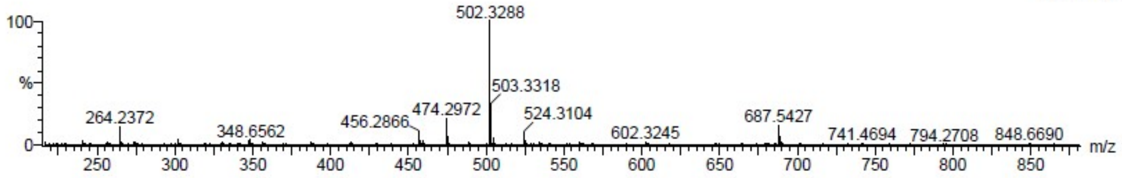

Minimum:				-1.5				
Maximum:		5.0	10.0	50.0				
Mass	Calc. Mass	mDa	PPM	DBE	i-FIT	Norm	Conf(%)	Formula
502.3288	502.3281	0.7	1.4	8.5	818.0	n/a	n/a	C28 H44 N3 O5

Figure S18: High resolution mass spectrum of compound **ABS-B4**.

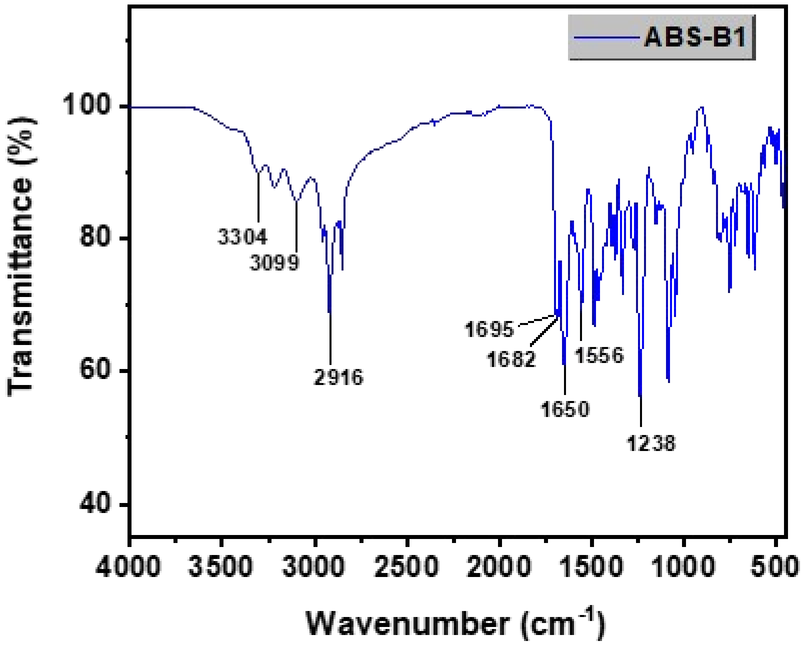

Figure S19: FT-IR spectrum of compound **ABS-B1**.

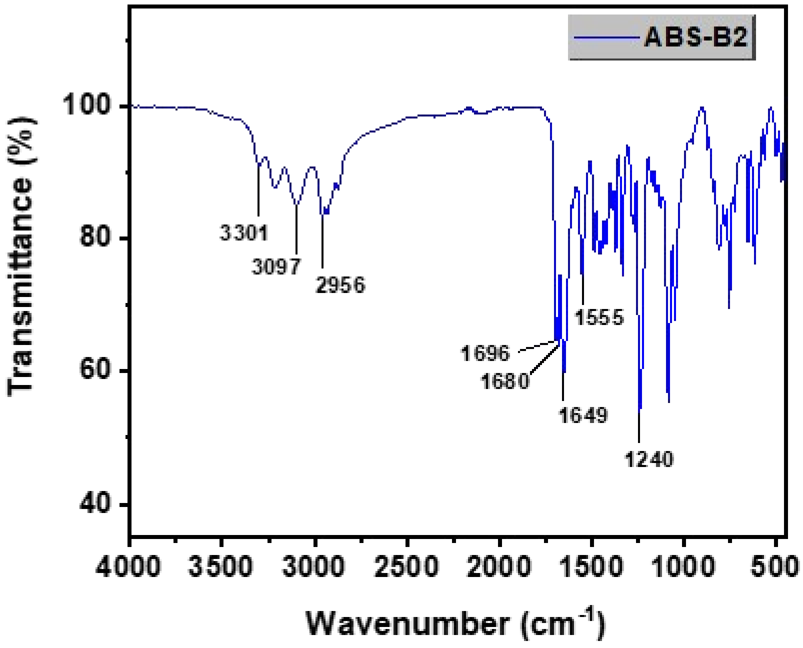

Figure S20: FT-IR spectrum of compound **ABS-B2**.

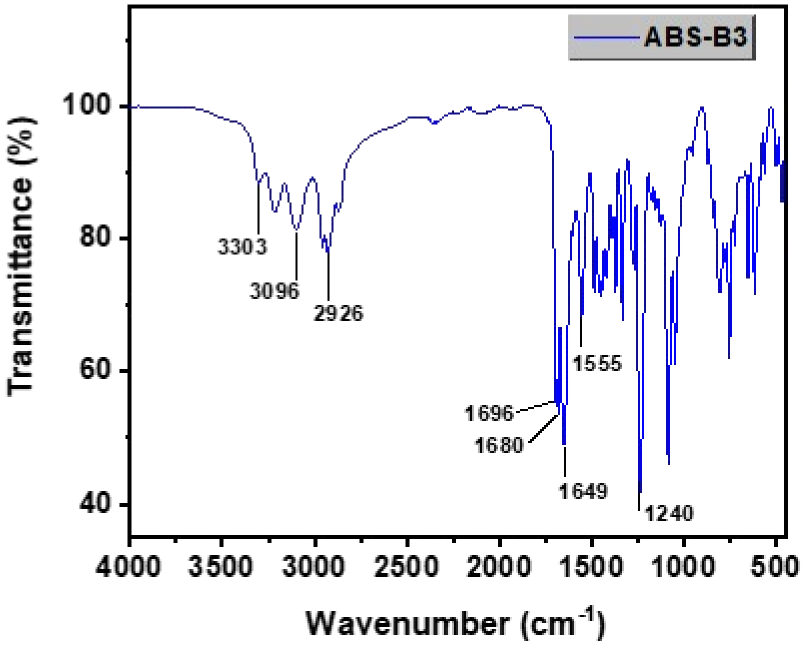

Figure S21: FT-IR spectrum of compound **ABS-B3**.

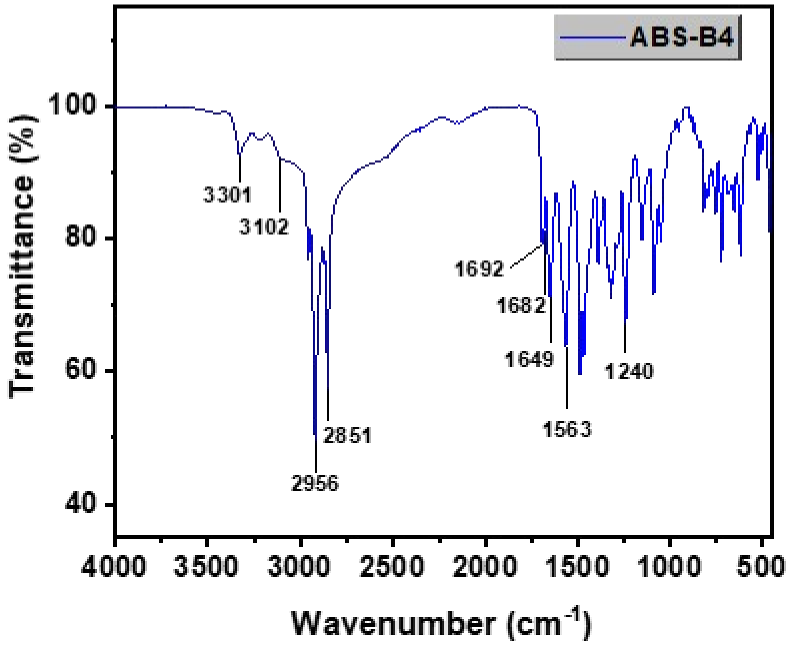

Figure S22: FT-IR spectrum of compound **ABS-B4**.
